# Supplementary material for: MK2 Phosphorylates RIPK1 to Prevent TNF-Induced Cell Death
Source: Mol Cell. 2017 Jun 1;66(5):698–710.e5. doi: 10.1016/j.molcel.2017.05.003 (PMC5459754; doi:10.1016/j.molcel.2017.05.003)
Supplement: Document S1. Figures S1–S6 [file mmc1.pdf]

Molecular Cell, Volume *66*

Supplemental Information

MK2 Phosphorylates RIPK1 to Prevent TNF-Induced Cell Death

Isabel Jaco, Alessandro Annibaldi, Najoua Lalaoui, Rebecca Wilson, Tencho Tenev, Lucie Laurien, Chun Kim, Kunzah Jamal, Sidonie Wicky John, Gianmaria Liccardi, Diep Chau, James M. Murphy, Gabriela Brumatti, Rebecca Feltham, Manolis Pasparakis, John Silke, and Pascal Meier

Supplementary Figures

Figure S1

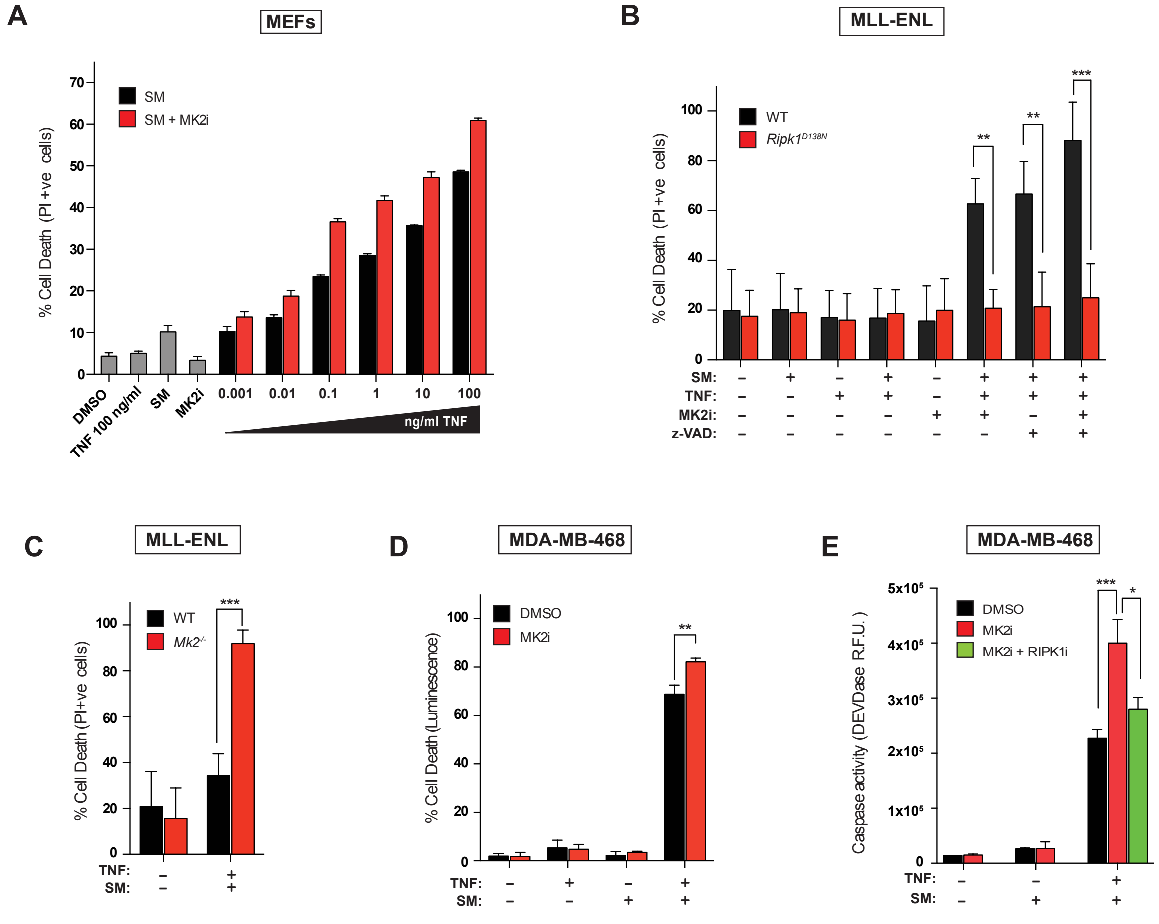

Figure S1. MK2 protects from TNF-induced cell death, Related to Figure 1

(A) Quantification of PI positive cells of primary MEFs treated with the indicated agents. Cells were pre-treated with MK2i (1 µM). **(B)** Quantification of PI positive cells of WT and *Ripk1*^{D138N} MLL-ENL cells. Cells were treated with the indicated agents for 24 hrs. TNF (10 ng/ml), SM (25 nM), MK2i (2 µM). **(C)** Quantification of PI positive cells of WT and *Mk2*$^{-/-}$ MLL-ENL cells, treated with the indicated agents for 24 hrs. TNF (10 ng/ml), SM (25 nM). **(D)** Quantification of cell viability in MDA-MB-468 cells, treated with the indicated agents. Cells were pre-treated with MK2i (1 µM). **(E)** DEVDase activity analysis of MDA-MB-468 cells left untreated or treated with the indicated agents for 5 hrs. Cells were pre-treated with MK2i (1 µM) and RIPK1i (100 nM) for 30 min. Graphs show mean ± SEM, n = 3 independent biological repeats. *p < 0.05, **p < 0.01, and ***p < 0.001.

Figure S2

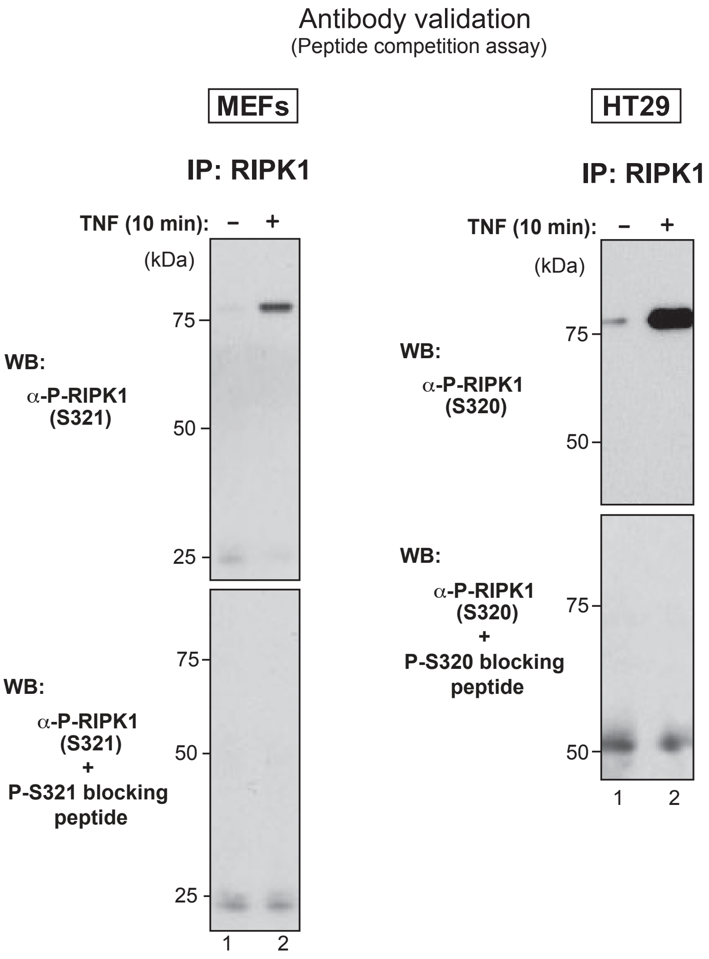

Figure S2. Antibody validation, Related to Figure 2

Western blot analysis of RIPK1 immunoprecipitates from MEFs or HT29 cells using the indicated antibodies. Cells were stimulated with TNF (10 ng/ml) for 10 min. Antibodies were either left untreated or pre-incubated with blocking P-peptide, against which these antibodies were raised against.

Figure S3

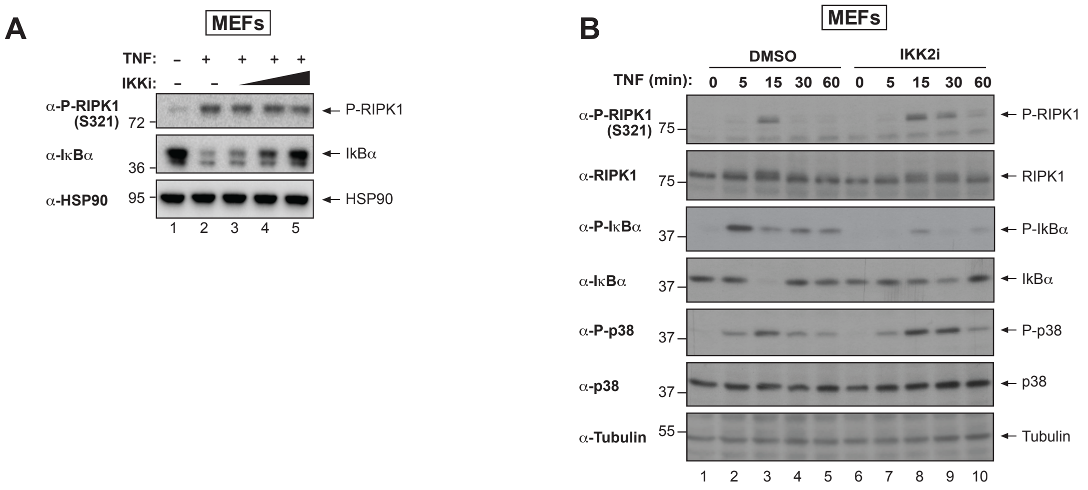

Figure S3. MK2 phosphorylates RIPK1 at S320/S321 in response to TNF stimulation, Related to Figure 3

(A) Western blot analysis of cell lysates from primary MEFs using the indicated antibodies. Cells were pre-treated with increasing concentrations of IKK2i (TCPA-1, 0.2, 1 and 5 μM) for 1 h, followed by stimulation with TNF (10 ng/ml) for 10 min. **(B)** Western blot analysis of cell lysates from primary MEFs using the indicated antibodies. Cells were pre-treated with IKK2i (BI605906, 10 μM) for 1 hr, followed by stimulation with TNF (10 ng/ml) for the indicated times.

Figure S4

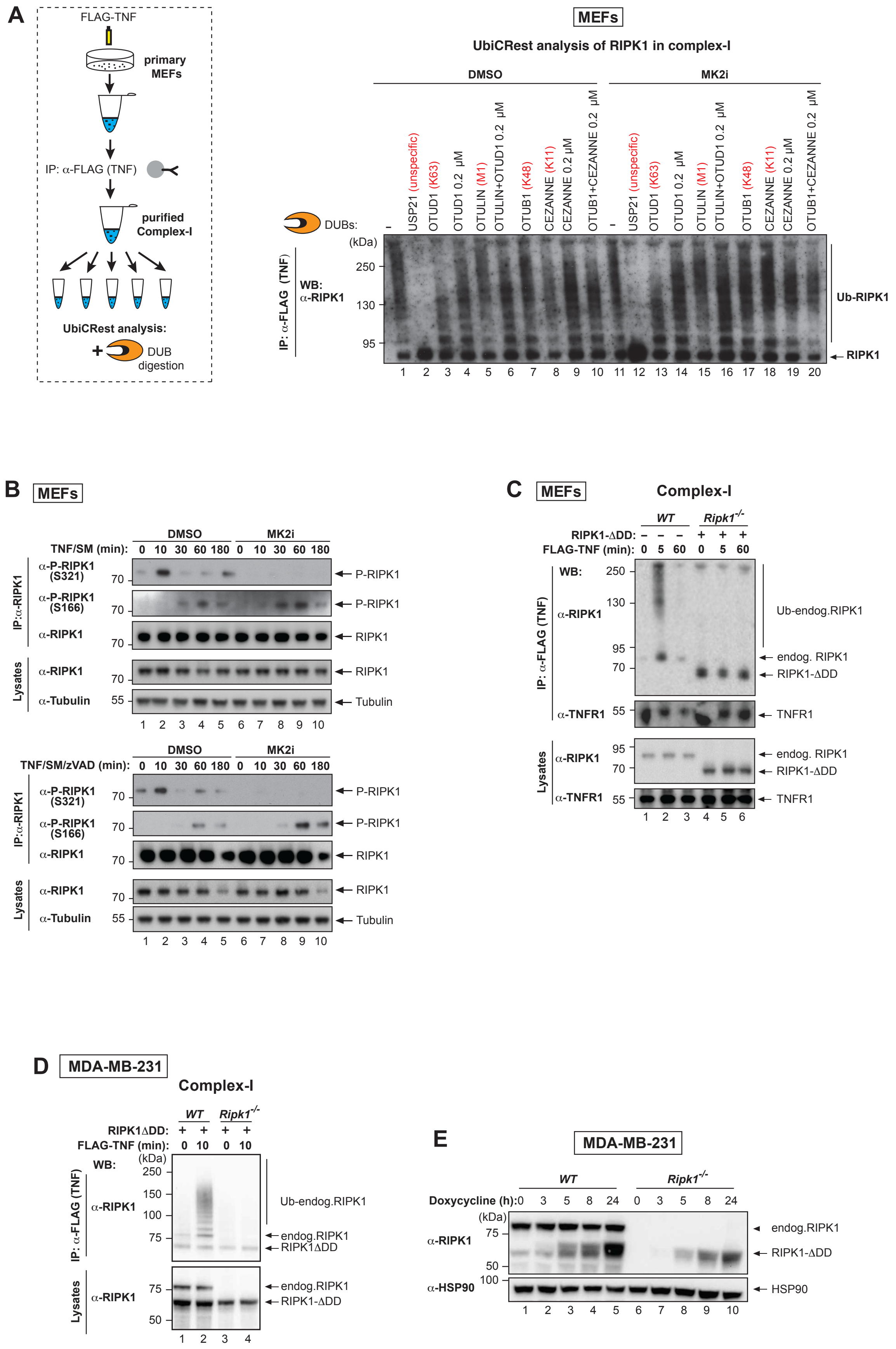
A
FLAG-TNF
primary MEFs
IP: α-FLAG (TNF)
purified Complex-I
UbiCRest analysis:
DUB digestion
MEFs
UbiCRest analysis of RIPK1 in complex-I
DMSO
MK2i
DUBs:
USP21 (unspecific)
OTUD1 (K63)
OTUD1 0.2 µM
OTULIN (M1)
OTULIN+OTUD1 0.2 µM
OTUB1 (K48)
CEZANNE (K11)
CEZANNE 0.2 µM
OTUB1+CEZANNE 0.2 µM
IP: α-FLAG (TNF)
WB: α-RIPK1
Ub-RIPK1
RIPK1
B
MEFs
TNF/SM (min):
TNF/SM/zVAD (min):
α-P-RIPK1 (S321)
α-P-RIPK1 (S166)
α-RIPK1
α-Tubulin
IP:α-RIPK1
Lysates
P-RIPK1
RIPK1
Tubulin
C
MEFs
Complex-I
WT
Ripk1-/-
RIPK1-ΔDD:
FLAG-TNF (min):
α-TNFR1
Ub-endog.RIPK1
endog. RIPK1
RIPK1-ΔDD
TNFR1
D
MDA-MB-231
Complex-I
RIPK1ΔDD:
FLAG-TNF (min):
E
MDA-MB-231
Doxycycline (h):
α-HSP90
HSP90

Figure S4. MK2-dependent phosphorylation of RIPK1 does not affect NF-κB signalling, Related to Figure 4

(A) UbiCRest analysis of ubiquitylated RIPK1 in complex-I. Complex-I was purified from primary WT MEFs using FLAG-mTNF as affinity reagent. Immuno-complexes were then subjected to UbiCRest analysis using the indicated panel of DUBs followed by Western blot analysis for RIPK1. **(B)** Immuno-precipitation of RIPK1 from DMSO and MK2i-treated MEFs (1 μM). The presence of the indicated proteins was evaluated by western blot. Cells were treated with the indicated agents for the indicated time points. **(C,D)** TNF-induced complex-I immuno-precipitation using FLAG-mTNF or FLAG-hTNF (1 μg /ml) as an affinity reagent from lysates of WT and *Ripk1*$^{-/-}$ MEFs (C) and MDA-MB-231 (D) cells stably expressing an inducible form of RIPK1-ΔDD. Western blot analysis with the indicated antibodies is shown. **(E)** Western blot analysis of cell lysates of WT and *Ripk1*$^{-/-}$ MDA-MB-231 cells stably expressing RIPK1-ΔDD. Expression of RIPK1-ΔDD was induced by Doxycycline (1 μg/ml), and the presence and phosphorylation status of the indicated proteins was evaluated.

Figure S5

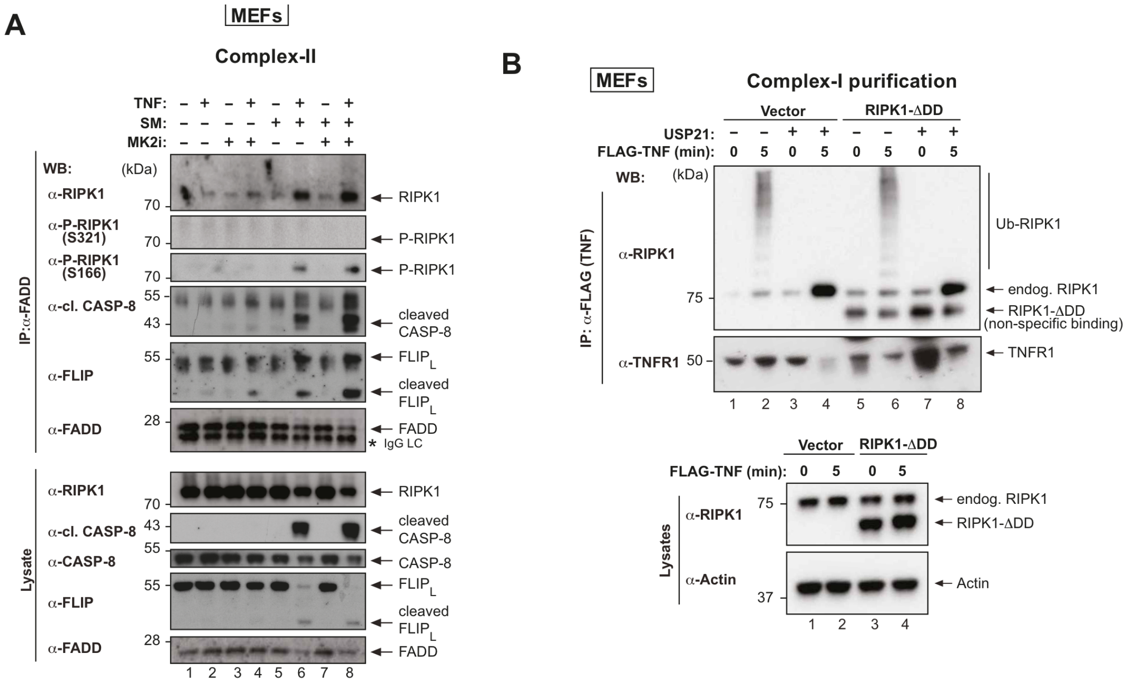

Figure S5. RIPK1-ΔDD is not recruited to TNF complex-I, Related to Figure 5

(A) TNF-induced complex-II was immuno-precipitated with anti-FADD from MEF lysates. Cells were treated with TSZ for 3 hrs ± MK2i (2 μM). **(B)** TNF-induced complex-I immuno-precipitation using FLAG-mTNF (1 μg/ml) as an affinity reagent from lysates of WT MEFs stably expressing and inducible form of RIPK1-ΔDD. Western blot analysis with the indicated proteins is shown.

Figure S6

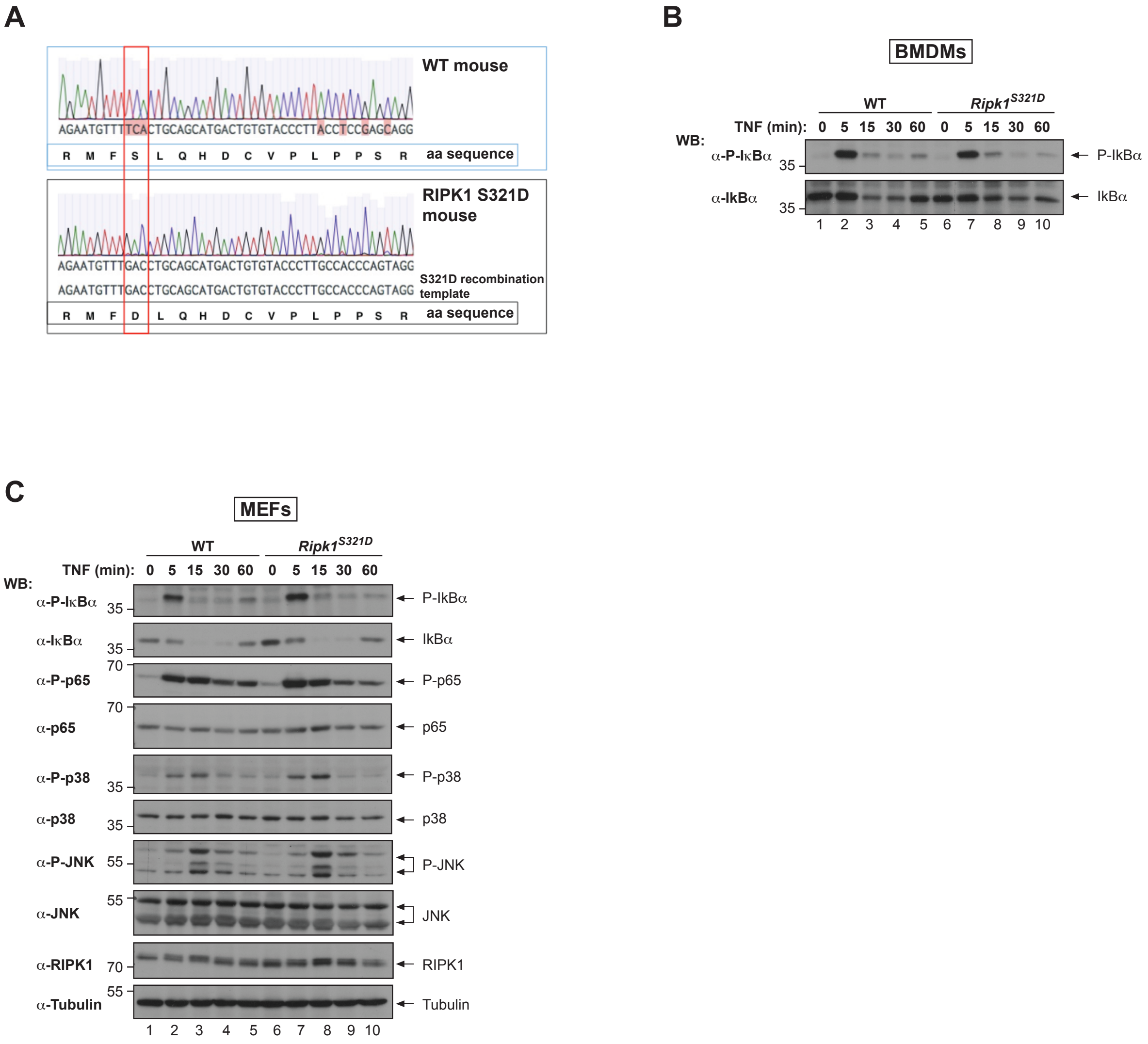

Figure S6. MK2-dependent phosphorylation of RIPK1 at S321 protects cells from TNF induced cell death, Related to Figure 6

(A) Sequence of the *Ripk1* gene in WT and *Ripk1*$^{S321D/S321D}$ mice. In addition to the TCA to GAC mutation changing S at position 321 to D, silent mutations were introduced in the PAM sequence to protect the mutated allele from Cas9-mediated cleavage. **(B, C)** Western blot analysis of primary BMDMs (B) and MEFs (C) isolated from WT and *Ripk1*^{S321D} mice using the indicated antibodies. Cells were treated with TNF (10 ng/ml) for the indicated time points.
