# Supplementary material for: MK2 Phosphorylates RIPK1 to Prevent TNF-Induced Cell Death
Source: Mol Cell. 2017 Jun 1;66(5):698–710.e5. doi: 10.1016/j.molcel.2017.05.003 (PMC5459754; doi:10.1016/j.molcel.2017.05.003)
Supplement: Document S2. Article plus Supplemental Information [file mmc2.pdf]

Molecular Cell

Article

MK2 Phosphorylates RIPK1 to Prevent TNF-Induced Cell Death

Graphical Abstract

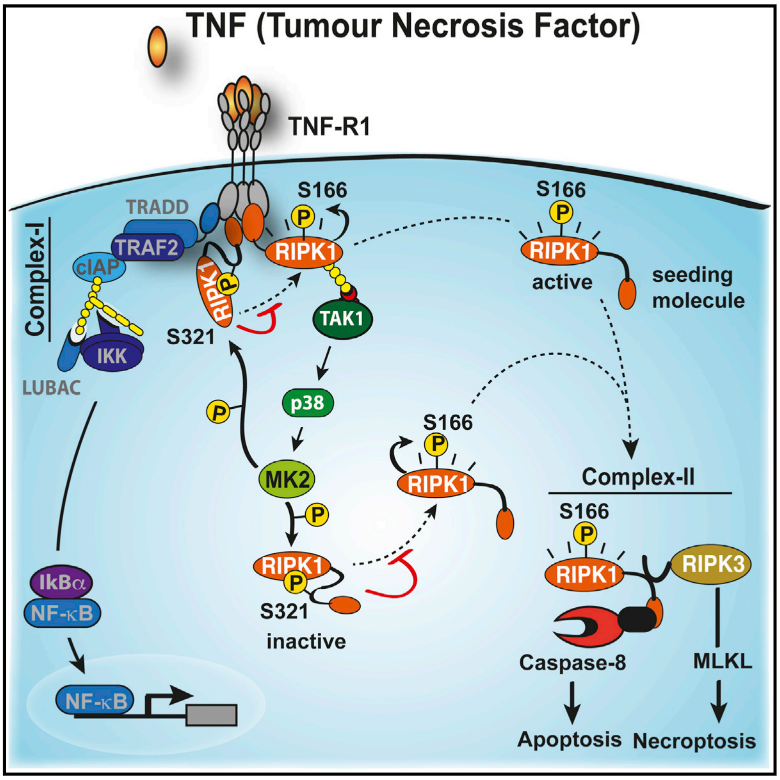

Highlights

- Phosphorylation of RIPK1 by MK2 acts as survival checkpoint in TNF signaling
- TNF-induced activation of MK2 results in global RIPK1 phosphorylation
- MK2-mediated phosphorylation suppresses RIPK1 kinase activation and cell death
- Complex-II originates from RIPK1 in complex-I as well as cytosolic RIPK1

Authors

Isabel Jaco, Alessandro Annibaldi, Najoua Lalaoui, ..., Manolis Pasparakis, John Silke, Pascal Meier

Correspondence

pasparakis@uni-koeln.de (M.P.),
silke@wehi.edu.au (J.S.),
pmeier@icr.ac.uk (P.M.)

In Brief

Jaco et al. show that MK2 directly phosphorylates RIPK1 at residue S321, suppressing the cytotoxic potential of RIPK1 and acting as a checkpoint within the TNF signaling pathway.

Jaco et al., 2017, Molecular Cell 66, 698–710
July 1, 2017 © 2017 The Author(s). Published by Elsevier Inc.
http://dx.doi.org/10.1016/j.molcel.2017.05.003

CellPress

Molecular Cell
Article

MK2 Phosphorylates RIPK1 to Prevent TNF-Induced Cell Death

Isabel Jaco,[1,7] Alessandro Annibaldi,[1,7] Najoua Lalaoui,[2,3,7] Rebecca Wilson,[1,7] Tencho Tenev,[1] Lucie Laurien,[4,5,6] Chun Kim,[4,5,6] Kunzah Jamal,[1] Sidonie Wicky John,[1] Gianmaria Liccardi,[1] Diep Chau,[2,3] James M. Murphy,[2,3] Gabriela Brumatti,[2,3] Rebecca Feltham,[1,2,3] Manolis Pasparakis,[4,5,6,8,*] John Silke,[2,3,8,*] and Pascal Meier[1,8,9,*]

[1]Breast Cancer Now Toby Robins Research Centre, Institute of Cancer Research, Mary-Jean Mitchell Green Building, Chester Beatty Laboratories, Fulham Road, London SW3 6JB, UK
[2]Walter and Eliza Hall Institute of Medical Research, Parkville, VIC 3052, Australia
[3]Department of Medical Biology, University of Melbourne, Parkville, VIC 3050, Australia
[4]Institute for Genetics
[5]Centre for Molecular Medicine (CMMC)
[6]Cologne Excellence Cluster on Cellular Stress Responses in Aging-Associated Diseases (CECAD)
University of Cologne, 50931 Cologne, Germany
[7]These authors contributed equally
[8]Senior author
[9]Lead Contact
*Correspondence: pasparakis@uni-koeln.de (M.P.), silke@wehi.edu.au (J.S.), pmeier@icr.ac.uk (P.M.)
http://dx.doi.org/10.1016/j.molcel.2017.05.003

SUMMARY

TNF is an inflammatory cytokine that upon binding to its receptor, TNFR1, can drive cytokine production, cell survival, or cell death. TNFR1 stimulation causes activation of NF-κB, p38α, and its downstream effector kinase MK2, thereby promoting transcription, mRNA stabilization, and translation of target genes. Here we show that TNF-induced activation of MK2 results in global RIPK1 phosphorylation. MK2 directly phosphorylates RIPK1 at residue S321, which inhibits its ability to bind FADD/caspase-8 and induce RIPK1-kinase-dependent apoptosis and necroptosis. Consistently, a phospho-mimetic S321D RIPK1 mutation limits TNF-induced death. Mechanistically, we find that phosphorylation of S321 inhibits RIPK1 kinase activation. We further show that cytosolic RIPK1 contributes to complex-II-mediated cell death, independent of its recruitment to complex-I, suggesting that complex-II originates from both RIPK1 in complex-I and cytosolic RIPK1. Thus, MK2-mediated phosphorylation of RIPK1 serves as a checkpoint within the TNF signaling pathway that integrates cell survival and cytokine production.

INTRODUCTION

Tumor necrosis factor (TNF) is a major inflammatory cytokine that was first identified for its ability to induce rapid hemorrhagic necrosis of cancers (Balkwill, 2009). In response to insults and infection, TNF contributes to homeostasis by regulating inflammation, cell proliferation, differentiation, survival, and death (Walczak, 2011). However, excessive or chronic engagement of TNFR1 can result in inflammatory diseases. Originally, it was considered that TNF contributes to such diseases by directly inducing the expression and production of inflammatory cytokines. However, recent evidence suggests that aberrant TNF-induced cell death may also contribute to the disease pathology (Gerlach et al., 2011; Pasparakis and Vandenabeele, 2015; Silke et al., 2015).

There are a number of different mechanisms to regulate TNF-induced cell death, including the formation of two distinct signaling complexes (Micheau and Tschopp, 2003). Within minutes of stimulation, TNFR1 assembles complex-I by recruiting the adaptors TRADD and TRAF2, the kinase RIPK1, and the E3 ubiquitin (Ub) ligases cellular inhibitor of apoptosis 1 (cIAP1) and cIAP2 (Silke, 2011; Ting and Bertrand, 2016). cIAPs subsequently conjugate various types of Ub linkages to components of this complex, which in turn allows Ub-dependent recruitment of the kinase complex TAK1/TAB2/TAB3 and the E3 ligase linear Ub chain assembly complex (LUBAC, composed of HOIL-1/HOIP/Sharpin). LUBAC-mediated linear ubiquitylation of different components of this complex appears to stabilize or reinforce complex-I formation and promote TAK1-dependent activation of IKK2. Formation of complex-I causes activation of NF-κB and mitogen-activated protein kinases (MAPKs), which ultimately results in the production of cytokines and pro-survival proteins, such as cFLIP, that are necessary for a coordinated inflammatory response (Elliott et al., 2016; Hrdinka et al., 2016; Kupka et al., 2016; Schlicher et al., 2016; Silke, 2011; Wagner et al., 2016).

TNF also initiates formation of an RIPK1-based cytoplasmic complex that chronologically appears after complex-I, and which can induce cell death. Therefore, this complex is frequently referred to as complex-II or the necrosome (Pasparakis and Vandenabeele, 2015; Wang et al., 2008). Complex-II can kill by activating caspase-8 and apoptosis, or via RIPK3 and MLKL, which results in necroptosis. It is currently believed that a small fraction of RIPK1 dissociates from complex-I within

 © 2017 The Author(s). Published by Elsevier Inc.
This is an open access article under the CC BY license (http://creativecommons.org/licenses/by/4.0/).

30 min to 3 hr, and together with TRADD, associates with the adaptor protein FADD and procaspase-8 to form complex-II (Micheau and Tschopp, 2003). Whether TNF can induce lethal levels of complex-II is dependent on multiple checkpoints: cIAP- and LUBAC-mediated ubiquitylation of RIPK1 are decisive factors in limiting complex-II formation (Bertrand et al., 2008; Gerlach et al., 2011; Haas et al., 2009). In the absence of either cIAPs or LUBAC, TNF fails to activate canonical NF-κB effectively, and consequently, cFLIP levels are insufficient to prevent caspase-8-mediated cell death. Under normal conditions, $cFLIP_L$ suppresses TNF-induced cell death by heterodimerizing with caspase-8. This inhibits formation of complex-II and the necrosome by cleaving RIPK1, RIPK3, and CYLD (Feng et al., 2007; Lin et al., 1999; O'Donnell et al., 2011; Oberst et al., 2011).

TAK1 and IKK2 also inhibit TNF-induced cell death. This has mainly been considered to be via induction of NF-κB and cFLIP; however, recent evidence suggests that they also regulate TNF killing independently of their role in NF-κB activation (Dondelinger et al., 2015; Kondylis et al., 2015; O'Donnell et al., 2007; Vlantis et al., 2016). In the absence of functional TAK1 or IKK, lethal levels of complex-II assemble despite RIPK1 ubiquitylation in complex-I (Dondelinger et al., 2013, 2015; Legarda-Addison et al., 2009). Under these conditions, TNF-mediated, RIPK1-dependent apoptosis was shown to rely on the kinase activity of RIPK1 (Dondelinger et al., 2013; Wang et al., 2008). It is unclear, however, whether TAK1 inhibits RIPK1 kinase activity directly, or indirectly via downstream kinases such as IKK2 (Dondelinger et al., 2015).

MAPK14 (p38α) and its substrate MAPKAPK2 (MK2) play essential roles in TNF-induced inflammatory cytokine production. Consequently, several pharmaceutical compounds have been developed to target these kinases in auto-inflammatory diseases (Genovese, 2009). However, recently we proposed that the p38-MK2 axis also regulates TNF- and RIPK1-dependent SMAC-mimetic (SM)-induced cell death (Lalaoui et al., 2016). These results therefore suggest that TAK1 mediates its pro-survival effect, at least in part, through activation of p38-MK2 (Sakurai, 2012).

While it is now thought that many chronic inflammatory diseases are caused or exacerbated by aberrant cytokine-induced cell death, the molecular events that regulate this process are largely unknown. In this study, we demonstrate that RIPK1 is a bona fide substrate of MK2 in both human and mouse. We find that TNF-induced activation of MK2 selectively protects cells from RIPK1 kinase-dependent death. While MK2-mediated phosphorylation of RIPK1 at S321 (mouse) and S320 (human) has no effect on NF-κB activation, it selectively inhibits RIPK1 kinase-mediated formation of complex-II, induction of apoptosis, and necroptosis. Whereas loss of S321 phosphorylation sensitizes cells to TNF killing, introduction of an S321 to D phospho-mimetic knockin mutation partly protects from RIPK1-dependent cell death upon TNF stimulation. We find that MK2-mediated phosphorylation of RIPK1 at S321/S320 inhibits RIPK1 kinase activation. We further show that cytosolic RIPK1 contributes to complex-II-mediated cell death, independent of its recruitment to complex-I, suggesting that complex-II originates from both RIPK1 in complex-I and cytosolic RIPK1. Our data demonstrate that the TAK1 > p38 > MK2 kinase cascade directly limits the lethal potential of cytosolic and complex-I-associated RIPK1, thereby licensing TNF-induced transcription, mRNA stabilization, and increased translation of cytokines necessary for a coordinated inflammatory response.

RESULTS

MK2 Protects from TNF-Induced Cell Death

We have shown that inhibition of p38α, or its downstream kinase MK2, enhances the killing activity of the SM birinapant (Lalaoui et al., 2016). Because SM kills cells by increasing the production of SM-induced TNF biosynthesis, and sensitizing cells to TNF-induced and RIPK1-mediated cell death, p38/MK2 might influence the sensitivity to TNF by influencing either or both of these processes. To distinguish between these scenarios, we treated bone marrow-derived macrophages (BMDMs) with SM and increasing concentrations of exogenous TNF and found that inhibition of MK2 sensitized BMDMs to TNF/SM (TS)-induced cell death in a dose-dependent manner, already 3 hr after treatment (Figure 1A). This suggests that inhibition of MK2 can sensitize cells to SM-induced killing independently of its role in inducing TNF biosynthesis (Gaestel, 2016). To explore this further, we used primary mouse embryonic fibroblasts (MEFs) that do not produce autocrine TNF in response to SM (Vince et al., 2007) and hence are resistant to SM, caspase activation, and cell death (Figures 1B and 1C). Inhibition of MK2 sensitized primary MEFs to TS-induced caspase activation and cell death (Figures 1B, 1C, and S1A), and co-treatment with the RIPK1 kinase inhibitor GSK'963 (RIPK1i) reversed this sensitization (Figure 1C). Inhibition of MK2 also sensitized MEFs and human HT29 cells to RIPK1-dependent, TNF-induced necroptosis (Figures 1D and 1E). Consistent with the notion that the kinase activity of RIPK1 is required for TNF-induced cell death under these conditions, we found that primary MEFs and murine leukemic MLL-ENLs that express kinase dead RIPK1 were largely protected from TSM (TNF, SM, and MK2i)-induced death (Figures 1F and S1B). To exclude a potential off-target effect of the MK2i PF-3644022, we generated murine leukemic MLLENL $Mk2^{-/-}$ and found that the absence of MK2 highly sensitized those cells to TS-induced cell death (Figure S1C). MK2i also sensitized human breast cancer BT549 and MDA-MB-468 cells to TS (Figures 1G, 1H, S1D, and S1E), implying that MK2 inhibition sensitizes to TNF-induced cell death in general.

MK2 Directly Phosphorylates RIPK1 at S320/S321 in Response to TNF Stimulation

Recent quantitative mass spectrometry analyses have identified TNF-induced phosphorylation of S320 of human RIPK1 (Degterev et al., 2008; Krishnan et al., 2015). Intriguingly, the motif surrounding S320 of human RIPK1 is evolutionarily conserved and conforms to the phosphorylation consensus motif of MK2, which is defined as Φ-X-R-X-(L/N)-pS/T-(I/V/F/L)-X, where Φ is a bulky hydrophobic residue (Figure 2A) (Cargnello and Roux, 2011). We therefore hypothesized that MK2 phosphorylates the serine within this conserved motif, and raised phospho-specific antibodies against P-S320 of human and P-S321 of mouse RIPK1 (Figure S2). Consistent with the notion that RIPK1 is phosphorylated at this motif by MK2, we found that TNF treatment of

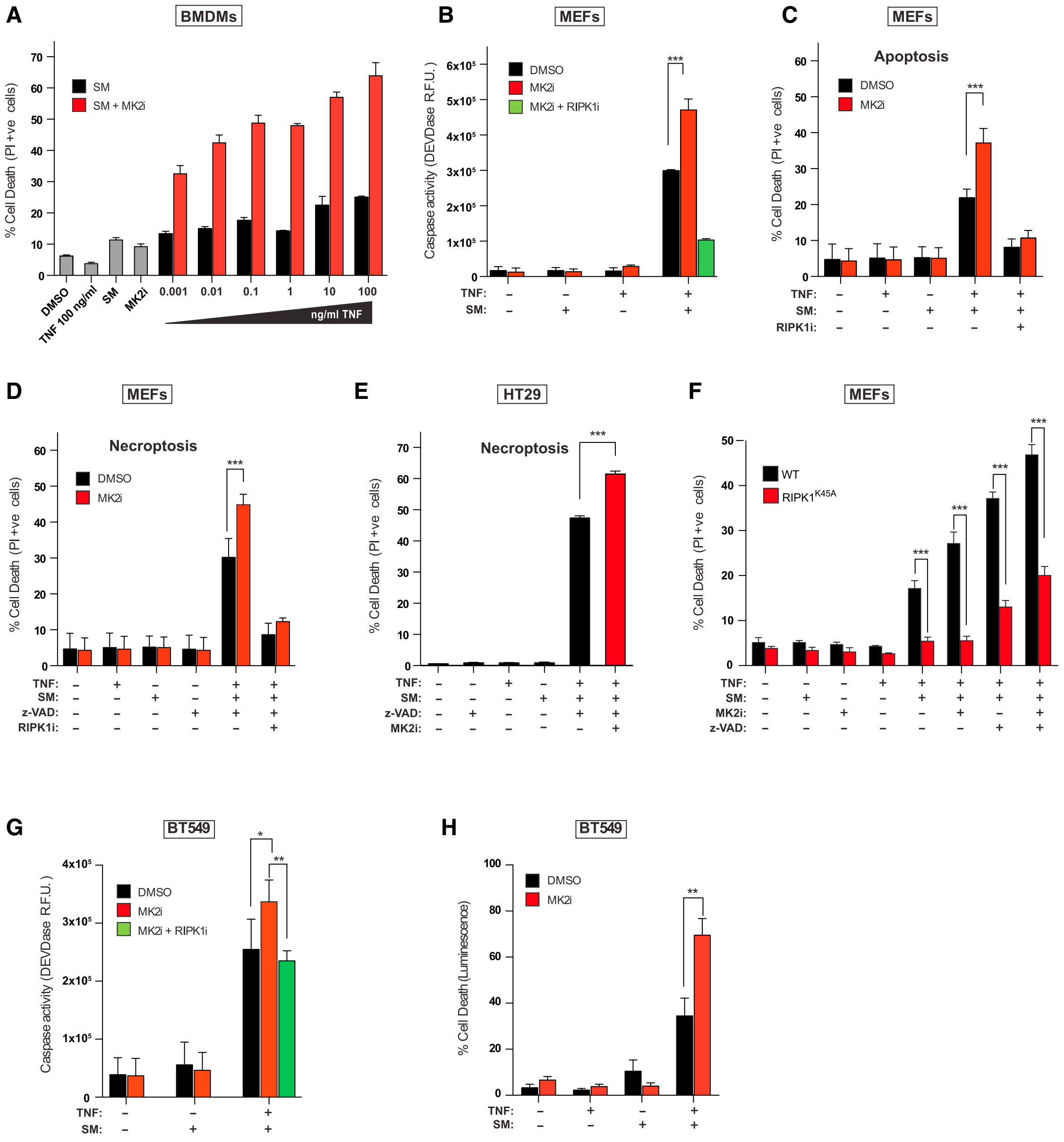

Figure 1. MK2 Protects from TNF-Induced Cell Death

(A) Quantification of PI-positive primary BMDMs treated with the indicated reagents for 3 hr. An early time point was chosen to avoid complications due to autocrine production of TNF. Cells were pre-treated with MK2i (1 μM) for 30 min.

(B) DEVDase activity analysis of primary MEFs treated with the indicated reagents for 4 hr. Cells were pre-treated with DMSO, MK2i (1 μM), or RIPK1i (100 nM) for 30 min.

(C) Quantification of PI-positive primary MEFs treated with the indicated reagents for 7 hr. Cells were pre-treated with MK2i or RIPK1i for 30 min.

(D–H) The indicated cells were treated with the respective reagents, and PI-positive (D–F and H) cells or DEVDase activity (G) was quantified. Cells were pre-treated with MK2i and/or RIPK1i for 30 min. Graphs show mean ± SEM, n = 3–5 independent repeats. $^{*}p < 0.05$, $^{**}p < 0.01$, and $^{***}p < 0.001$.

See also Figure S1.

primary MEFs resulted in transient phosphorylation of RIPK1 at S321, which was blocked by pharmacological inhibition or genetic deletion of MK2 (Figures 2B and 2C). We found that phosphorylated RIPK1 migrates differently depending on the gel type used and was readily distinguishable from the un-phosphorylated form when lysates were separated on an 8% gel (Figure 2C). Similarly, TNF treatment induced RIPK1 phosphorylation of S321 in primary BMDMs in an MK2-dependent manner (Figure 2D). Likewise, human RIPK1 was phosphorylated at S320 in MDA-MB-468 cells (Figure 2E).

MK2 is activated by p38α in response to many stimuli, including cytokines and bacterial infection (Cargnello and

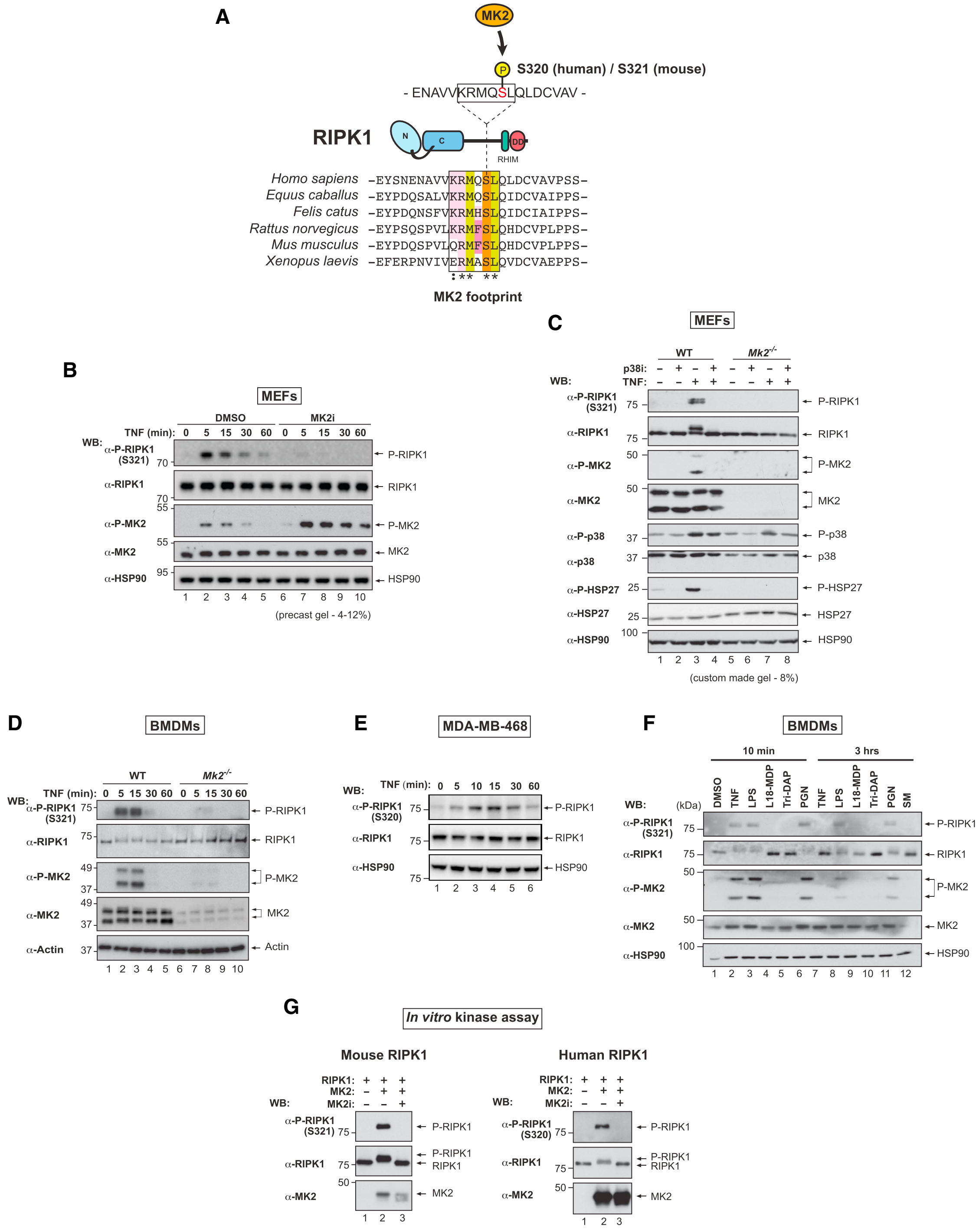

Figure 2. MK2 Directly Phosphorylates RIPK1 at S320/S321 in Response to TNF Stimulation

(A) Schematic depicting the evolutionarily conserved MK2 phosphorylation consensus sequence of RIPK1. Color scheme emphasizes sequence conservation within the motif.

(B) Western blot analysis of cell lysates separated on a 4%–12% gradient gel from primary MEFs using the indicated antibodies. Cells were pre-treated with DMSO or MK2i (1 μM, 30 min), followed by treatment with TNF (10 ng/mL) for the indicated time points.

(C) Western blot analysis of cell lysates separated on a Tris-glycine 8% gel from primary WT or *Mk2*$^{-/-}$ MEFs using the indicated antibodies. Cells were pre-treated with DMSO or p38i (1 μM, 30 min), followed by a 10 min treatment with TNF (10 ng/mL).

(D) Western blot analysis of cell lysates from WT or *Mk2*$^{-/-}$ BMDMs using the indicated antibodies. Cells were treated ± TNF (10 ng/mL) for the indicated times.

(E) Western blot analysis of protein lysates from MDA-MB-468 cells using the indicated antibodies. Cells were treated ± TNF (10 ng/mL) for the indicated times.

(legend continued on next page)

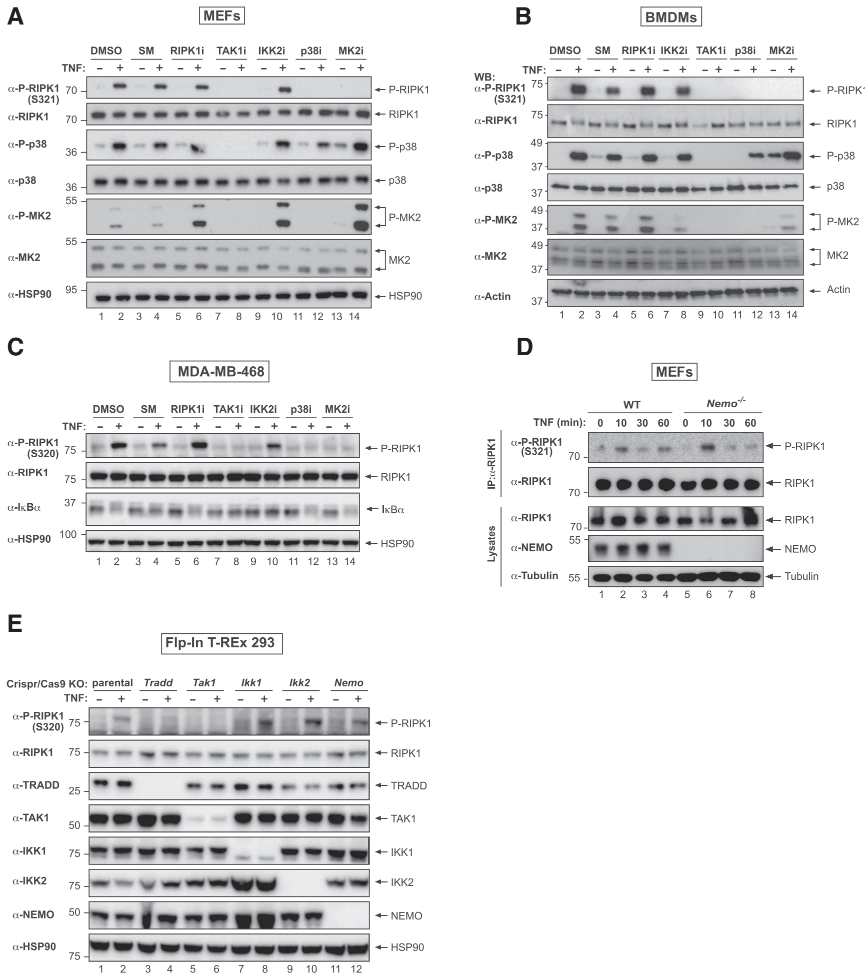

Figure 3. Phosphorylation of RIPK1 at S320/321 Is Dependent on the TAK1 > p38α > MK2 Signaling Cascade but Independent of IKK

(A) Western blot analysis of cell lysates of the indicated cells using the described antibodies. Cells were left untreated or pre-treated for 30 min with the indicated inhibitors, followed by TNF treatment (10 ng/mL, 10 min) (SM CompA, 500 nM; RIPK1i GSK'963, 100 nM; TAK1i (5Z)-7-O, 1 μM; IKKi TPCA-1, 5 μM; p38i, 1 μM; MK2i PF3644022, 1 μM).

(B) Western blot analysis of cell lysates from BMDMs subjected to pre-treatment for 30 min with the indicated inhibitors (SM CompA, 500 nM; RIPK1i/Nec1s, 1 μM; TAK1i (5Z)-7-O, 250 nM; IKKi TPCA-1, 250 nM; p38i/LY2228820, 250 nM; MK2i/PF3644022, 2 μM), followed by treatment with TNF (100 ng/mL) for 10 min.

(C) Western blot analysis of cell lysates from BMDMs subjected to pre-treatment for 30 min with the indicated inhibitors, followed by TNF treatment (10 ng/mL, 10 min), as in (A).

(D) Western blot analysis of cell lysates from immortalized WT and *Nemo*$^{-/-}$ MEFs treated with TNF for the indicated time points.

(E) Western blot analysis of cell lysates from Flp-In T-REx 293 cells in which the respective genes were knocked out using CRISPR/Cas9. Cells were treated with human TNF (10 ng/mL) for 10 min.

See also Figure S3.

Roux, 2011). Consistent with the idea that RIPK1 is phosphorylated by MK2, stimuli that activated MK2, as measured by the appearance of phospho-MK2 (P-T222), also lead to phosphorylation of RIPK1 S321 (Figure 2F). LPS- and PGN-induced phosphorylation of S321 was longer lasting than the one triggered by TNF. To determine whether MK2 directly phosphorylated RIPK1, we conducted an in vitro kinase assay using recombinant MK2 and purified RIPK1. MK2 readily phosphorylated mouse and human RIPK1 on S320 and S321, respectively (Figure 2G).

Phosphorylation of RIPK1 at S320/321 Is Dependent on the TAK1 > p38α > MK2 Signaling Cascade but Independent of IKK

To dissect the signaling cascade that results in RIPK1 phosphorylation at S320/S321, we made use of pharmacologic inhibition and genetic mutation of components of the TNF receptor signaling complex. Phosphorylation of RIPK1 at S320/321 was dependent on the TAK1-p38α-MK2 kinase cascade because inhibition of either TAK1 or p38α, which block TNF-induced MK2 phosphorylation and activation (Figures 3A–3C), or inhibition of MK2 itself, abolished the appearance of P-S321 in primary MEFs and BMDMs, and of P-S320 in human breast cancer MDA-MB-468 cells (Figures 3A–3C). While pharmacological inhibition of IKK2 with TPCA-1 or BI605906 strongly inhibited IκBα degradation, as expected (Figures S3A and S3B), it did not prevent S320/321 phosphorylation in any of the three cell types tested (Figures 3A–3C, S3A, and S3B). Likewise, genetic deletion of NEMO, IKK1, or IKK2 did not interfere with TNF-induced phosphorylation of S320/S321 in mouse and human cells (Figures 3D and 3E). The IKK complex, therefore, does not appear to be involved in mediating phosphorylation of RIPK1 at these residues. Furthermore, treatment with an RIPK1 inhibitor did not interfere with S320/321 phosphorylation following TNF stimulation (Figures 3A–3C), implying that P-S320/321 is not an auto-phosphorylation event.

(F) Western blot analysis of cell lysates separated on Tris-glycine 8% gel BMDMs using the indicated antibodies. Cells were stimulated with the indicated reagents.

(G) In vitro kinase assay using purified proteins. Recombinant active human MK2 was incubated with mouse and human RIPK1 in the presence of DMSO or MK2i and the reactions separated on a Tris-glycine 8% acrylamide gel. The presence of phosphorylated S321/320 RIPK1 and MK2 was evaluated using the indicated antibodies.

See also Figure S2.

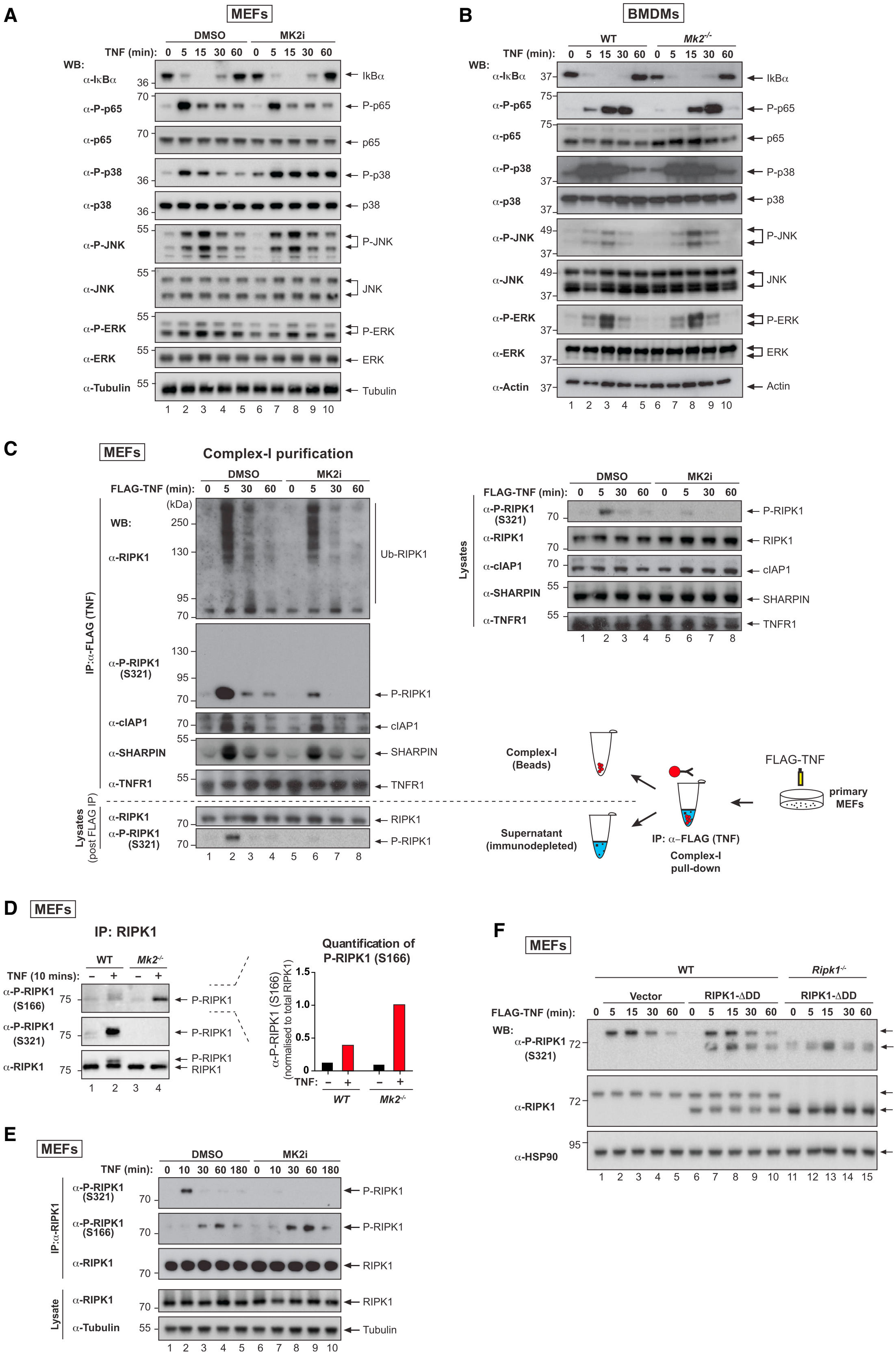

(legend on next page)

MK2-Dependent Phosphorylation of RIPK1 Inhibits RIPK1 Activation but Does Not Impede TNF-Induced Activation of NF-κB

Binding of TNF to TNFR1 results in activation of NF-κB and MAPKs, leading to transcriptional induction of pro-inflammatory cytokines as well as pro-survival genes such as cFLIP and cIAPs. Since defects in NF-κB are known to sensitize cells to TNF-induced cell death (Peltzer et al., 2016), we examined whether inhibition of MK2 affected TNF-induced activation of NF-κB and MAPK. However, inhibition or deletion of MK2 had no effect on TNF-induced degradation of IκBα or phosphorylation of p65, JNK, or ERK in MEFs and BMDMs (Figures 4A and 4B). Moreover, we found no evidence for defective ubiquitylation of RIPK1 in complex-I (Figure 4C), and UbiCRest (Ub chain restriction) analysis (Hospenthal et al., 2015) of ubiquitylated RIPK1 in complex-I revealed no qualitative differences in Ub linkage types in the presence or absence of MK2i (Figure S4A). Intriguingly, only the non-ubiquitylated form of RIPK1 in complex-I was phosphorylated at S321 (Figure 4C). In contrast, phosphorylation at S166 of RIPK1 in complex-I readily occurs on ubiquitylated RIPK1 (Newton et al., 2016). Further, we found that RIPK1 was significantly more phosphorylated on S166 in *Mk2*$^{-/-}$ cells or in cells treated with MK2 inhibitors in response to TNF (Figures 4D and 4E), although the timing of S166 phosphorylation was unaffected by MK2 inhibition (Figures 4E and S4B). We found that P-S166 appeared after P-S321. Of note, the kinetics of P-S321 did not appear to change with SM, which prevents ubiquitylation of RIPK1, or SM+zVAD, which in addition inhibits caspases (Figure S4B). Together, these results suggest that MK2-mediated RIPK1 S321 phosphorylation occurs in an IAP- and Ub modification-independent manner. While P-S321 RIPK1 in complex-I is not ubiquitylated, this phosphorylation does not prevent normal levels of ubiquitylated RIPK1 from being generated in this complex. Further, our data support the notion that P-S321 suppresses RIPK1 S166 auto-phosphorylation.

Remarkably, P-S321 RIPK1 was present in both complex-I and the complex-I immuno-depleted fraction (lysates post-FLAG immunoprecipitation) after only 5 min of TNF stimulation (Figure 4C), suggesting that cytosolic RIPK1 is phosphorylated by MK2. To conclusively test whether recruitment of RIPK1 to complex-I was dispensable for S321 phosphorylation, we reconstituted wild-type (WT) and *Ripk1*$^{-/-}$ MEFs with an RIPK1 mutant that lacks the death domain (ΔDD). This mutant is not recruited to complex-I and, therefore, cannot become ubiquitylated (Figures S4C–S4E). Even though RIPK1-ΔDD was not recruited to complex-I, it was readily phosphorylated at S321 (Figure 4F). Together, these data demonstrate that TNF activates MK2, which in turn rapidly phosphorylates non-ubiquitylated RIPK1 in complex-I and the cytosol.

MK2 Limits Complex-II Formation

Thus far, our data suggest that MK2 inhibition neither affects TNF-induced recruitment of RIPK1 into complex-I nor limits activation of NF-κB/MAPK pathways, yet increases phosphorylation of RIPK1 on S166 and sensitizes cells to TNF-induced death. This, therefore, suggests a role for MK2 in regulating RIPK1 and complex-II formation. Consistent with this, we found that loss of MK2 dramatically enhanced TNF-induced association of RIPK1, FADD, and active caspase-8 (Figure 5A). Pharmacological inhibition of MK2 similarly increased complex-II formation and activation in response to TS (Figures 5B and S5A).

These data suggested that more RIPK1 was available for recruitment into complex-II and prompted us to monitor the levels of ubiquitylated RIPK1 in the presence and absence of active MK2 post-TNF stimulation. Using tandem Ub binding entities (TUBEs) (Hjerpe et al., 2009), which allow isolation of polyubiquitylated proteins, we purified all ubiquitylated proteins over a TNF time course and probed with an anti-RIPK1 antibody. Using the non-specific deubiquitylating enzyme (DUB), USP21, to confirm ubiquitylation, we found that in WT cells, the levels of ubiquitylated RIPK1 increased within 15 min of TNF stimulation, and then steadily decreased over 3 hr of TNF treatment (Figure 5C). Upon MK2 inhibition, the levels of ubiquitylated RIPK1 were more prominent at the earliest times following TNF stimulation. TNF-induced accumulation of RIPK1 in the ubiquitylated fraction correlated with a significant increase in formation of complex-II and activation of caspase-8 (Figures 5A, 5B, and S5A).

The observation that cytosolic RIPK1 is phosphorylated by MK2 within minutes of TNF stimulation (Figures 4C and 4D) raises the question of the "origin" of complex-II. Complex-II may be assembled from RIPK1 that (1) comes entirely from complex-I, (2) is generated from the cytosolic pool of RIPK1, or (3) is seeded by RIPK1 from complex-I and augmented by cytosolic RIPK1. To test this, we reconstituted WT and *Ripk1*$^{-/-}$ MEFs with RIPK1-ΔDD that retains the homotypic RHIM oligomerization domain and hence can form functional amyloid signaling complexes (Li et al., 2012). RIPK1-ΔDD-expressing *Ripk1*$^{-/-}$ MEFs were as resistant as *Ripk1*$^{-/-}$ MEFs to TS-induced cell death (Figure 5D), demonstrating that the cytosolic pool of RIPK1 on its own is unable to stimulate cell death in response to TS either in the presence or absence of MK2i. However, RIPK1-ΔDD exacerbated TNF killing when endogenous WT RIPK1 was present, even

Figure 4. MK2-Dependent Phosphorylation of RIPK1 Does Not Affect NF-κB Signaling but Suppresses RIPK1 Activation

(A) Western blot analysis of cell lysates from primary MEFs using the indicated antibodies. Cells were treated with TNF (10 ng/mL) for the indicated time points.
(B) Western blot analysis of cell lysates from WT or *Mk2*$^{-/-}$ BMDMs using the indicated antibodies. Cells were treated with TNF (100 ng/mL) for the indicated time points.
(C) TNF-induced complex-I immunoprecipitation. Primary MEFs were treated with FLAG-mTNF (1 μg/mL) for the indicated time points, followed by FLAG immunoprecipitation and western blot analysis. Lysates pre- (right) and post-immunoprecipitation (bottom) were also analyzed by western blot.
(D) Immunoprecipitation of RIPK1 from WT and *Mk2*$^{-/-}$ immortalized MEFs, treated ± TNF (10 ng/mL). A Tris-glycine 8% acrylamide gel was used to visualize the RIPK1 phospho-dependent mobility shift. Quantification of the intensity of the P-S166 signal, normalized to total RIPK1, is shown to the right.
(E) Immunoprecipitation of RIPK1 from MEFs treated with TNF (10 ng/mL) ± MK2i (1 μM). The presence of the indicated proteins was evaluated by western blot.
(F) WT and *Ripk1*$^{-/-}$ MEFs stably expressing murine RIPK1-ΔDD were stimulated with FLAG-mTNF (1 μg/mL) for the indicated time points. Western blot analysis with the indicated antibodies is shown.
See also Figure S4.

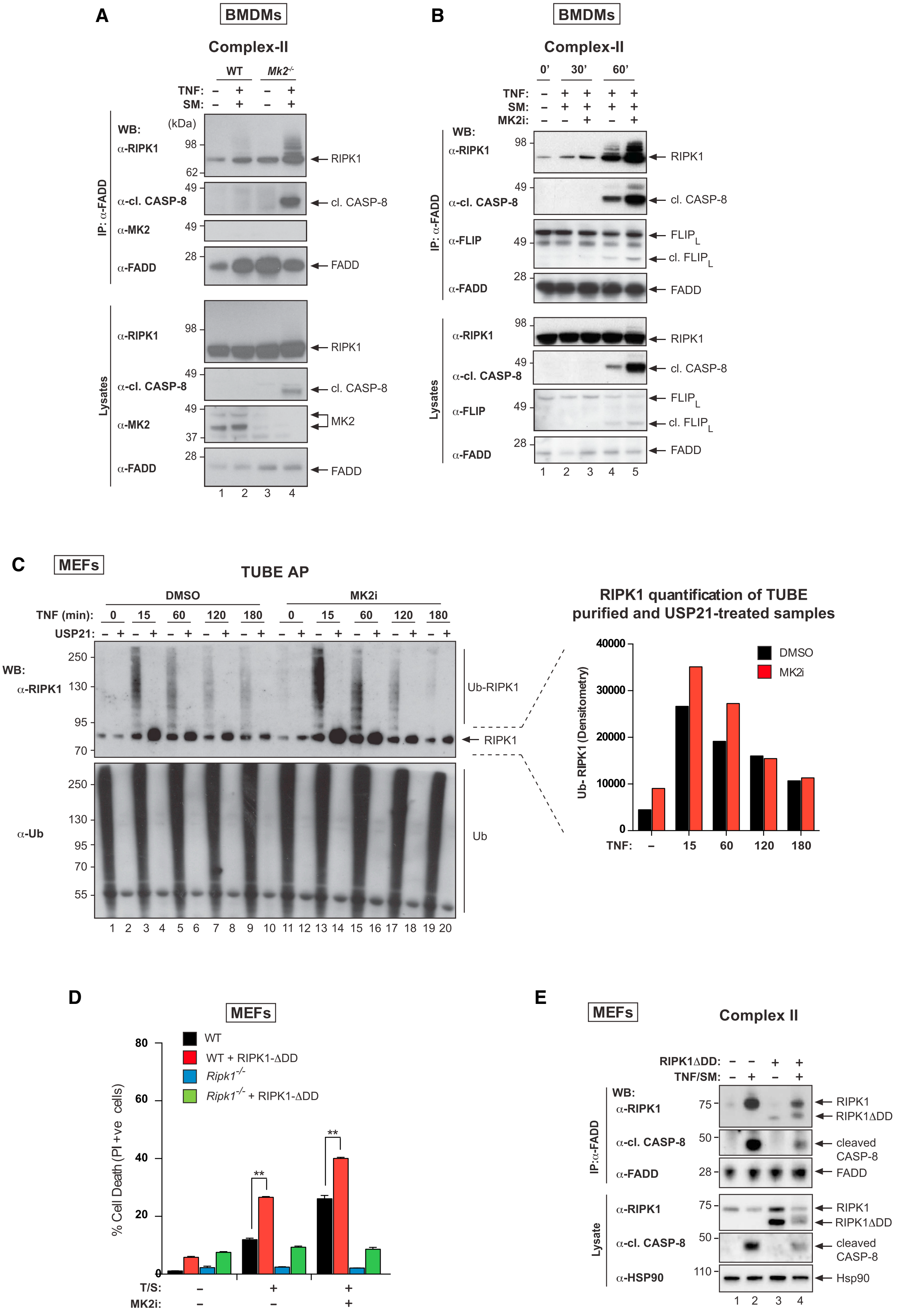

(legend on next page)

though RIPK1-ΔDD is not recruited to complex-I following TNF treatment (Figures S4C, S4D, and S5B). Inhibition of MK2 further enhanced this death (Figure 5D). Consistent with the notion that RIPK1-ΔDD is directly recruited to complex-II, we found that it co-purified with components of complex-II in response to TS (Figure 5E). Together, these data suggest that the cytosolic pool of RIPK1 can contribute to complex-II and cell death and does not need to be first recruited to complex-I.

MK2-Dependent Phosphorylation of RIPK1 at S321 Protects Cells from TNF-Induced Cell Death

To examine the importance of phosphorylation at S321, we generated RIPK1 S321D phospho-mimetic knockin mice using CRISPR/Cas9 technology (Figure S6A). RIPK1 S321D mice were born and weaned at the expected Mendelian ratio (data not shown) and were indistinguishable from their WT littermates. Primary MEFs from RIPK1 S321D animals exhibited the same RIPK1 protein levels, indicating that the S321D mutation had no impact on the stability of RIPK1 (Figure S6C). TNF-induced activation of NF-κB and MAP kinases in MEFs and BMDMs from these mice was also indistinguishable from WT cells (Figures S6B and S6C), consistent with our observations that inhibition or deletion of MK2 had no effect on TNF-induced NF-κB/MAPK activation. However, BMDMs and MEFs of homozygous S321D animals were less sensitive to TS-induced apoptosis and caspase activation compared to their WT littermate controls (Figures 6A–6C). The protective effect of the S321D mutation was lost at later time points, suggesting that this phosphorylation event delays, but cannot prevent, TNF-induced cell death. Moreover, given that inhibition of MK2 sensitizes S321D cells, it is likely that MK2 phosphorylates additional sites on RIPK1. To examine whether S321D cells have a lower propensity to form complex-II using an independent method, we performed an in situ proximity ligation assay (PLA) (Söderberg et al., 2006) with a combination of RIPK1 and caspase-8 antibodies that generate a localized, discrete signal only when RIPK1 and caspase-8 are in a complex (Orme et al., 2016). Compared to littermate WT MEFs, *Ripk1*^{S321D} MEFs were significantly less efficient in forming complex-II (Figure 6D). Together, our data demonstrate that phosphorylation of S321 by MK2 protects from RIPK1-mediated cell death.

DISCUSSION

TNF is a major inflammatory cytokine that was first identified for its ability to induce rapid hemorrhagic necrosis of cancers (Balkwill, 2009). While TNF can cause cell death, the dominant outcome in most cell types is cell survival and the production of pro-inflammatory cytokines. Several checkpoints control TNF-induced and RIPK1-dependent cell death (O'Donnell and Ting, 2011). In this study, we identified a new checkpoint that limits death induced by TNF when cIAPs are limiting, which can occur when cells become stressed by cytotoxic agents (Tenev et al., 2011; Yang et al., 2000) or as a result of signaling from other TNF receptor super family members (Feoktistova et al., 2011; Vince et al., 2008). Mechanistically, TNF induces phosphorylation of RIPK1 on a serine embedded within an evolutionarily conserved MK2 consensus sequence. RIPK1 phosphorylation at S320 (human) or S321 (mouse) by MK2 suppresses TS-induced cell death. Genetic deletion or pharmacological inhibition of MK2 prevents this phosphorylation and, thereby, enhances TNF-driven and RIPK1-dependent cell death. Although the importance of this survival checkpoint is revealed when cIAPs are limiting, we found that TNF and other inflammatory ligands are also potent inducers of RIPK1 phosphorylation in several different cell types, suggesting that MK2-mediated regulation of RIPK1 may be a more general phenomenon.

TNF/TNFR1 induces at least two cellular signaling complexes (Micheau and Tschopp, 2003): the initial receptor-associated plasma membrane complex (complex-I) that activates NF-κB and MAPK, and hence transcription and translation, and a secondary cytosolic complex (complex-II) whose role appears to be to initiate cell death. Whether complex-I is connected with complex-II, and if so, how and in what manner it contributes to the formation of complex-II, remains unclear (Silke, 2011). TNF induces RIPK1 and cIAP recruitment to the TNFR1 receptor to generate complex-I in which RIPK1 and other components of complex-I are rapidly ubiquitylated by cIAPs. The conjugation of Ub to RIPK1 and components of complex-I (Wong et al., 2010) promotes TAK1-mediated activation of IKK2, JNK, ERK, and p38α. p38α phosphorylates and activates MK2, which is known to phosphorylate substrates that regulate mRNA stability (Gurgis et al., 2015). Phosphorylation of RIPK1 on S321 by MK2 is an early and transient event in TNF signaling as it occurs within 5 min and is lost after 30 min. While RIPK1 in complex-I is phosphorylated at S321 within minutes, a large proportion of the cytosolic pool of RIPK1 is also rapidly phosphorylated by MK2. How MK2 is able to rapidly access and phosphorylate this pool of RIPK1 is an intriguing question, and prompted us to explore its relevance. Whereas loss of NF-κB signaling can sensitize cells to TNF-induced death, we were unable to find any defects in

Figure 5. MK2 Limits Complex-II Formation

(A) TNF-induced complex-II immunoprecipitation using anti-FADD. Western blot analysis of complex-II from WT and *Mk2*$^{-/-}$ BMDMs using the indicated antibodies. Cells were treated with TNF (100 ng/mL) and SM (500 nM) for 1 hr and z-VAD-FMK (20 μM) to stabilize complex-II.

(B) TNF-induced complex-II was immunoprecipitated with anti-FADD from BMDM lysates. Cells were treated with TSZ (as in A) for the indicated times ± Mk2i (2 μM).

(C) TUBE affinity purification of lysates from primary MEFs. Cells were pretreated with DMSO or MK2i (1 μM) for 30 min, and treated ± TNF for the indicated times. The TUBE affinity-purified ubiquitylated proteome was subsequently left untreated or exposed to USP21. Western blot analysis for the indicated proteins is shown. The graph to the right depicts the quantification of non-modified RIPK1 in the USP21-treated samples.

(D) Quantification of PI-positive primary WT and *Ripk1*$^{-/-}$ MEFs reconstituted with RIPK1-ΔDD. Cells were pretreated with DMSO or MK2i (1 μM) for 30 min, followed by TS treatment for 3 hr. Graphs show mean ± SEM, n = 3 independent repeats. *p < 0.05, **p < 0.01, and ***p < 0.001.

(E) TNF-induced complex-II was immunoprecipitated with anti-FADD from lysates of WT MEFs reconstituted with RIPK1-ΔDD (as in D). Cells were treated with TSZ (as in A) for 3 hr.

See also Figure S5.

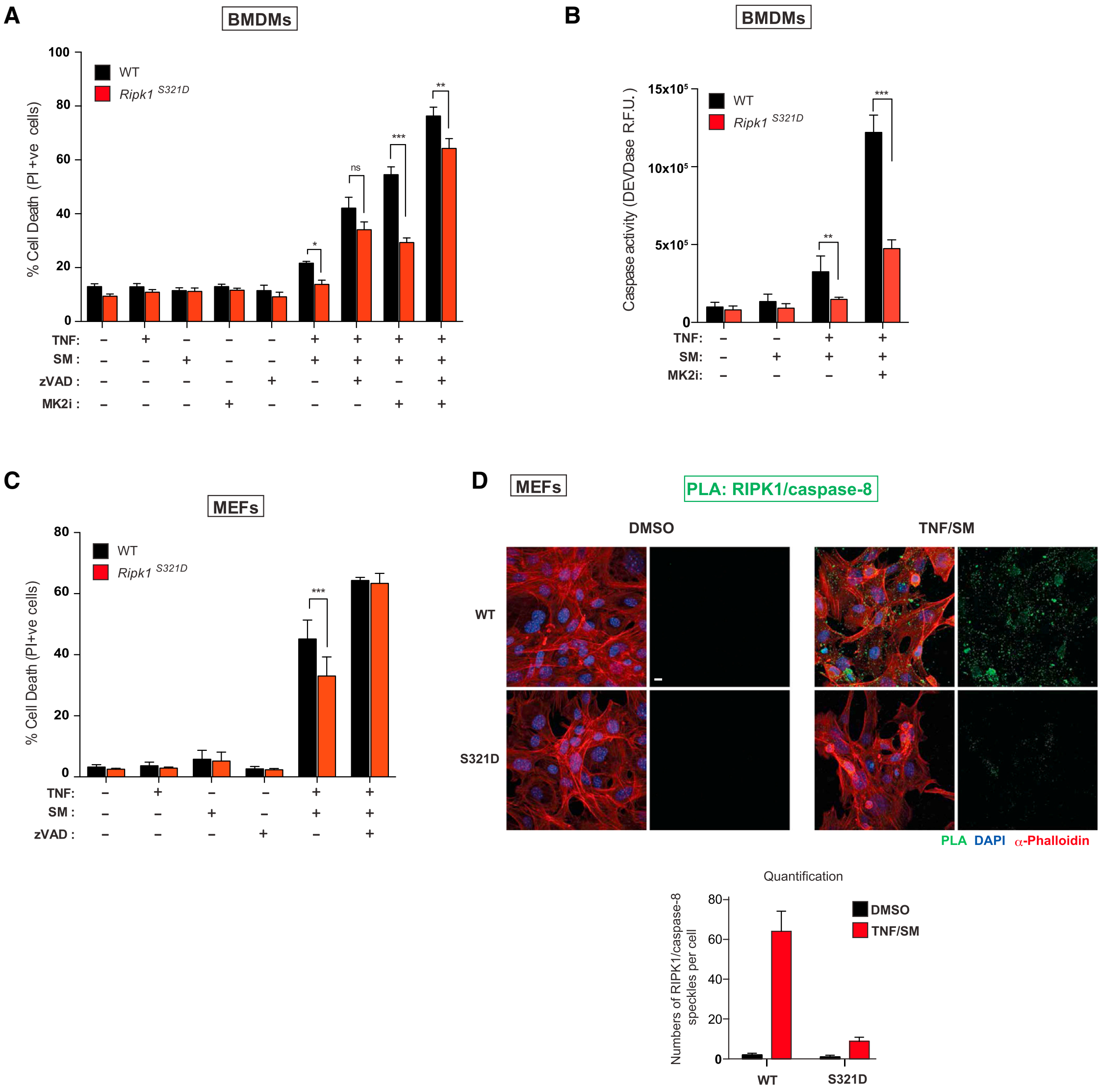

Figure 6. MK2-Dependent Phosphorylation of RIPK1 at S321 Protects Cells from TNF-Induced Cell Death

(A) Quantification of PI-positive WT and *Ripk1*^{S321D} BMDMs treated with the indicated reagents for 5 hr.

(B) DEVDase activity analysis of BMDMs treated with the indicated reagents for 1 hr.

(C) Quantification of PI-positive primary WT and *Ripk1*^{S321D} MEFs treated with the indicated reagents for 6 hr.

(D) PLA of primary WT and *Ripk1*^{S321D} MEFs using RIPK1 and caspase-8 antibodies. Cells were stimulated with the indicated reagents for 3 hr. The panel below shows quantifications of RIPK1/caspase-8 PLA speckles. Scale bar, 10 μm.

Graphs show mean ± SEM, n = 3–8 independent repeats. *p < 0.05, **p < 0.01, and ***p < 0.001.

See also Figure S6.

TNF-mediated RIPK1 ubiquitylation or NF-κB/MAPK activation in *Mk2*-deficient or MK2-inhibited cells. On the other hand, we found that in the absence of MK2, RIPK1 has a higher propensity to form complex-II. Recently, auto-phosphorylation of S166 in RIPK1 has been linked to its ability to induce cell death. Intriguingly, while RIPK1-P-S166 is readily ubiquitylated in complex-I (Newton et al., 2016), RIPK1-P-S321 is non-ubiquitylated in complex-I. Further, our time course analysis suggests that P-S321 may have to be removed before RIPK1's auto-activation at P-S166 can occur. While S321 phosphorylation may precede and/or preclude RIPK1 ubiquitylation in complex-I, it may also be possible that distinct pools of RIPK1 participate in S321 phosphorylation and ubiquitylation. In the latter case, RIPK1 phosphorylation at S321 may serve to limit the available pool of RIPK1 to be recruited to complex-I. Although these are attractive models, given that RIPK1 readily self-associates, it will be difficult to conclusively demonstrate whether P-S166 and P-S321 are mutually exclusive or compatible. Nevertheless, our results are consistent with a model whereby P-S321 antagonizes RIPK1 kinase auto-activation and RIPK1's killing activity. Consistently, we find that *Ripk1*$^{S321A/+}$ heterozygosity sensitizes primary mouse dermal fibroblasts to TS-induced cell death (N.L. and J.S., unpublished data).

MK2 not only phosphorylates RIPK1 in complex-I but also modifies a substantial pool of RIPK1 outside of this complex. Since complex-II assembles several hours after the formation of complex-I, we addressed the origin of the death-inducing platform. Using a form of RIPK1 that is not recruited to complex-I, we found that RIPK1 can be recruited to complex-II directly from the cytosolic pool. The recruitment of non-ubiquitylated, cytosolic RIPK1 directly to complex-II may help to explain why RIPK1 in complex-II predominantly lacks Ub chains, although undoubtedly deubiquitylating enzymes can also contribute to this phenomenon. Since MK2 is activated under various stress conditions that stimulate p38 (Cargnello and Roux, 2011), such as UV irradiation, heat shock, oxidative stress, hyperosmolarity, bacterial infection, and different cytokines, it is tempting to speculate that MK2 regulates RIPK1 under many of these stress conditions. While the p38 MAPK pathway is deregulated in all inflammatory diseases, p38 inhibitors have failed phase II clinical trials due to undesirable side effects (Duraisamy et al., 2008). It will be interesting to test whether some of these side effects may be due to deregulation of RIPK1.

We previously showed that inactivation of p38α or MK2 significantly improves SM-based therapeutic approaches, particularly in acute myeloid leukemia (AML). Accordingly, inhibition of these kinases greatly sensitized MLL-ENL-, MLL-, AF9-, NUP98-HoxA9-, and HoxA9/Meis1-expressing AML cells to killing by the clinical SM birinapant, in a TNFR1-dependent manner (Lalaoui et al., 2016). In these earlier experiments, loss or inhibition of p38α or MK2 rapidly increased birinapant-induced production of TNF by AML cells. Due to the rapid induction of TNF under these conditions, it was not practical to determine whether the enhanced sensitivity of AML cells was due to more TNF or heightened sensitivity to TNF killing, or both. However, the general nature of the results presented here makes it likely that p38/MK2 inhibition also sensitizes AML cells to TS death, and might help to account for the substantial in vivo efficacy of the SM/p38i combination treatment (Lalaoui et al., 2016). When SMs kill cells as single agents, as in AML cells, they do so via a two-pronged mechanism, simultaneously promoting TNF production and sensitizing to TNF- and RIPK1-dependent cell death (Varfolomeev et al., 2007; Vince et al., 2007; Wang et al., 2008; Wong et al., 2010). Thus, it is particularly intriguing that p38α/MK2 inhibition increases both facets of SM activity, suggesting a deeper connection between TNF production and TNF-induced cell death than previously anticipated. While the details of this link remain unclear, these new insights provide a further rationale for exploring the combined treatment of p38/MK2i and SM against cancers clinically (Wang, 2017).

STAR★METHODS

Detailed methods are provided in the online version of this paper and include the following:

- KEY RESOURCE TABLE
- CONTACT FOR REAGENT AND RESOURCE SHARING
- EXPERIMENTAL MODEL AND SUBJECT DETAILS
  - Experimental Model
  - Generation of murine AML
  - Mice generation
  - Cell lines
- METHOD DETAILS
  - Isolation of primary cells
  - Constructs and transfection
  - Cell death and cell viability assays
  - Caspase activity assay (DEVDase)
  - Generation of CRISPR cells
  - In vitro kinase assay
  - Tube pull-down
  - UbiCRest
  - Complex-I/II Purification
  - Proximity ligation assay (PLA)
- QUANTIFICATION AND STATISTICAL ANALYSIS
- DATA AND SOFTWARE AVAILABILITY

SUPPLEMENTAL INFORMATION

Supplemental Information includes six figures and can be found with this article online at http://dx.doi.org/10.1016/j.molcel.2017.05.003.

AUTHOR CONTRIBUTIONS

I.J. designed and performed experiments shown in Figures 1A–1E, 1G, 1H, 5D, 6A, S1A, S1D, S1E, and S6C. I.J. also wrote the paper, generated the figures, and coordinated the collaboration among the three laboratories. A.A. designed and performed the experiments shown in Figures 1F, 2B, 3A, 3D, 4A, 4C, 4E, 4F, 5C, 5D, S3A, S4A, S4B, and S5A. A.A. prepared MEFs and BMDMs used in Figures 1A–1D and generated RIPK1-ΔDD-expressing cells. R.W. discovered that MK2 phosphorylates RIPK1, and designed and performed the experiments shown in Figures 2C, 2F, 2G, 4D, and S2. N.L. designed and performed the experiments shown in Figures 2D, 3B, 4B, 5A, 5B, S1B, and S1C. T.T. designed and performed the experiments in Figures 2E, 3C, 3E, S4C, and S4D. T.T. also cloned and generated stable cell lines. K.J. performed the experiment shown in Figure S5. L.L. performed the experiments shown in Figures 3D, S3B, S6A, and S6B. C.K. designed and generated the RIPK1 S321D knockin mice. J.M.M., G.B., D.C., and R.F. performed pilot experiments. S.W.J. helped with cloning and the generation of CRISPR knockout cell lines. G.L. performed the experiments shown in Figure 6D. J.S., M.P., and P.M. designed and supervised the study and wrote the paper.

ACKNOWLEDGMENTS

We would like to thank M. Gaestel for tissue from *Mk2*$^{-/-}$ mice. We thank members of the Meier, Silke, and Pasparakis laboratories for helpful discussions and the CECAD Transgenic Core Facility for CRISPR/Cas9 mutagenesis in mouse zygotes. R.W., G.L., and T.T. are supported by Breast Cancer Now CTR-QR14-007. I.J. is supported by a World Wide Cancer Research grant (14-1328) and Breast Cancer Now CTR-QR14-007. A.A. is supported by the MRC grant MR/M019217/7. P.M. acknowledges NHS funding to the NIHR Biomedical Research Centre. M.P. acknowledges funding from the ERC (grant agreement no. 323040). N.L is supported by World Wide Cancer Research grant 15-0042. This work was funded by NHMRC grants 1025594, 1046984, 1081221, and 1081376 and was made possible through Victorian State Government Operational Infrastructure Support and Australian Government NHMRC IRIISS (9000220). J.S. is supported by fellowship 1107149 from the NHMRC. G.B. is supported by a Victoria Cancer Agency mid-career fellowship (VCA-MCRF15027). J.M.M. is supported by fellowship 1105754 from NHMRC. R.F. acknowledges funding from NHMRC 1081272.

Received: February 27, 2017
Revised: April 17, 2017
Accepted: May 3, 2017
Published: May 11, 2017

REFERENCES

Balkwill, F. (2009). Tumour necrosis factor and cancer. Nat. Rev. Cancer *9*, 361–371.

Berger, S.B., Kasparcova, V., Hoffman, S., Swift, B., Dare, L., Schaeffer, M., Capriotti, C., Cook, M., Finger, J., Hughes-Earle, A., et al. (2014). Cutting edge: RIP1 kinase activity is dispensable for normal development but is a key regulator of inflammation in SHARPIN-deficient mice. J. Immunol. *192*, 5476–5480.

Bertrand, M.J., Milutinovic, S., Dickson, K.M., Ho, W.C., Boudreault, A., Durkin, J., Gillard, J.W., Jaquith, J.B., Morris, S.J., and Barker, P.A. (2008). cIAP1 and cIAP2 facilitate cancer cell survival by functioning as E3 ligases that promote RIP1 ubiquitination. Mol. Cell *30*, 689–700.

Cargnello, M., and Roux, P.P. (2011). Activation and function of the MAPKs and their substrates, the MAPK-activated protein kinases. Microbiol. Mol. Biol. Rev. *75*, 50–83.

Choi, H., Larsen, B., Lin, Z.Y., Breitkreutz, A., Mellacheruvu, D., Fermin, D., Qin, Z.S., Tyers, M., Gingras, A.C., and Nesvizhskii, A.I. (2011). SAINT: probabilistic scoring of affinity purification-mass spectrometry data. Nat. Methods *8*, 70–73.

Degterev, A., Hitomi, J., Germscheid, M., Ch'en, I.L., Korkina, O., Teng, X., Abbott, D., Cuny, G.D., Yuan, C., Wagner, G., et al. (2008). Identification of RIP1 kinase as a specific cellular target of necrostatins. Nat. Chem. Biol. *4*, 313–321.

Dondelinger, Y., Aguileta, M.A., Goossens, V., Dubuisson, C., Grootjans, S., Dejardin, E., Vandenabeele, P., and Bertrand, M.J. (2013). RIPK3 contributes to TNFR1-mediated RIPK1 kinase-dependent apoptosis in conditions of cIAP1/2 depletion or TAK1 kinase inhibition. Cell Death Differ. *20*, 1381–1392.

Dondelinger, Y., Jouan-Lanhouet, S., Divert, T., Theatre, E., Bertin, J., Gough, P.J., Giansanti, P., Heck, A.J., Dejardin, E., Vandenabeele, P., and Bertrand, M.J. (2015). NF-κB-independent role of IKKα/IKKβ in preventing RIPK1 kinase-dependent apoptotic and necroptotic cell death during TNF signaling. Mol. Cell *60*, 63–76.

Duraisamy, S., Bajpai, M., Bughani, U., Dastidar, S.G., Ray, A., and Chopra, P. (2008). MK2: a novel molecular target for anti-inflammatory therapy. Expert Opin. Ther. Targets *12*, 921–936.

Elliott, P.R., Leske, D., Hrdinka, M., Bagola, K., Fiil, B.K., McLaughlin, S.H., Wagstaff, J., Volkmar, N., Christianson, J.C., Kessler, B.M., et al. (2016). SPATA2 links CYLD to LUBAC, activates CYLD, and controls LUBAC signaling. Mol. Cell *63*, 990–1005.

Feng, S., Yang, Y., Mei, Y., Ma, L., Zhu, D.E., Hoti, N., Castanares, M., and Wu, M. (2007). Cleavage of RIP3 inactivates its caspase-independent apoptosis pathway by removal of kinase domain. Cell. Signal. *19*, 2056–2067.

Feoktistova, M., Geserick, P., Kellert, B., Dimitrova, D.P., Langlais, C., Hupe, M., Cain, K., MacFarlane, M., Häcker, G., and Leverkus, M. (2011). cIAPs block Ripoptosome formation, a RIP1/caspase-8 containing intracellular cell death complex differentially regulated by cFLIP isoforms. Mol. Cell *43*, 449–463.

Gaestel, M. (2016). MAPK-activated protein kinases (MKs): novel insights and challenges. Front. Cell Dev. Biol. *3*, 88.

Genovese, M.C. (2009). Inhibition of p38: has the fat lady sung? Arthritis Rheum. *60*, 317–320.

Gerlach, B., Cordier, S.M., Schmukle, A.C., Emmerich, C.H., Rieser, E., Haas, T.L., Webb, A.I., Rickard, J.A., Anderton, H., Wong, W.W., et al. (2011). Linear ubiquitination prevents inflammation and regulates immune signalling. Nature *471*, 591–596.

Gurgis, F.M., Yeung, Y.T., Tang, M.X., Heng, B., Buckland, M., Ammit, A.J., Haapasalo, J., Haapasalo, H., Guillemin, G.J., Grewal, T., and Munoz, L. (2015). The p38-MK2-HuR pathway potentiates EGFRvIII-IL-1β-driven IL-6 secretion in glioblastoma cells. Oncogene *34*, 2934–2942.

Haas, T.L., Emmerich, C.H., Gerlach, B., Schmukle, A.C., Cordier, S.M., Rieser, E., Feltham, R., Vince, J., Warnken, U., Wenger, T., et al. (2009). Recruitment of the linear ubiquitin chain assembly complex stabilizes the TNF-R1 signaling complex and is required for TNF-mediated gene induction. Mol. Cell *36*, 831–844.

Hjerpe, R., Aillet, F., Lopitz-Otsoa, F., Lang, V., England, P., and Rodriguez, M.S. (2009). Efficient protection and isolation of ubiquitylated proteins using tandem ubiquitin-binding entities. EMBO Rep. *10*, 1250–1258.

Hospenthal, M.K., Mevissen, T.E., and Komander, D. (2015). Deubiquitinase-based analysis of ubiquitin chain architecture using Ubiquitin Chain Restriction (UbiCRest). Nat. Protoc. *10*, 349–361.

Hrdinka, M., Fiil, B.K., Zucca, M., Leske, D., Bagola, K., Yabal, M., Elliott, P.R., Damgaard, R.B., Komander, D., Jost, P.J., and Gyrd-Hansen, M. (2016). CYLD limits Lys63- and Met1-linked ubiquitin at receptor complexes to regulate innate immune signaling. Cell Rep. *14*, 2846–2858.

Kondylis, V., Polykratis, A., Ehlken, H., Ochoa-Callejero, L., Straub, B.K., Krishna-Subramanian, S., Van, T.M., Curth, H.M., Heise, N., Weih, F., et al. (2015). NEMO prevents steatohepatitis and hepatocellular carcinoma by inhibiting RIPK1 kinase activity-mediated hepatocyte apoptosis. Cancer Cell *28*, 582–598.

Krishnan, R.K., Nolte, H., Sun, T., Kaur, H., Sreenivasan, K., Looso, M., Offermanns, S., Krüger, M., and Swiercz, J.M. (2015). Quantitative analysis of the TNF-α-induced phosphoproteome reveals AEG-1/MTDH/LYRIC as an IKKβ substrate. Nat. Commun. *6*, 6658.

Kupka, S., De Miguel, D., Draber, P., Martino, L., Surinova, S., Rittinger, K., and Walczak, H. (2016). SPATA2-mediated binding of CYLD to HOIP enables CYLD recruitment to signaling complexes. Cell Rep. *16*, 2271–2280.

Lalaoui, N., Hänggi, K., Brumatti, G., Chau, D., Nguyen, N.Y., Vasilikos, L., Spilgies, L.M., Heckmann, D.A., Ma, C., Ghisi, M., et al. (2016). Targeting p38 or MK2 enhances the anti-leukemic activity of Smac-mimetics. Cancer Cell *29*, 145–158.

Legarda-Addison, D., Hase, H., O'Donnell, M.A., and Ting, A.T. (2009). NEMO/IKKgamma regulates an early NF-kappaB-independent cell-death checkpoint during TNF signaling. Cell Death Differ. *16*, 1279–1288.

Li, J., McQuade, T., Siemer, A.B., Napetschnig, J., Moriwaki, K., Hsiao, Y.S., Damko, E., Moquin, D., Walz, T., McDermott, A., et al. (2012). The RIP1/RIP3 necrosome forms a functional amyloid signaling complex required for programmed necrosis. Cell *150*, 339–350.

Lin, Y., Devin, A., Rodriguez, Y., and Liu, Z.G. (1999). Cleavage of the death domain kinase RIP by caspase-8 prompts TNF-induced apoptosis. Genes Dev. *13*, 2514–2526.

Mali, P., Yang, L., Esvelt, K.M., Aach, J., Guell, M., DiCarlo, J.E., Norville, J.E., and Church, G.M. (2013). RNA-guided human genome engineering via Cas9. Science *339*, 823–826.

Micheau, O., and Tschopp, J. (2003). Induction of TNF receptor I-mediated apoptosis via two sequential signaling complexes. Cell *114*, 181–190.

Newton, K., Wickliffe, K.E., Maltzman, A., Dugger, D.L., Strasser, A., Pham, V.C., Lill, J.R., Roose-Girma, M., Warming, S., Solon, M., et al. (2016). RIPK1 inhibits ZBP1-driven necroptosis during development. Nature *540*, 129–133.

O'Donnell, M.A., and Ting, A.T. (2011). RIP1 comes back to life as a cell death regulator in TNFR1 signaling. FEBS J. *278*, 877–887.

O'Donnell, M.A., Legarda-Addison, D., Skountzos, P., Yeh, W.C., and Ting, A.T. (2007). Ubiquitination of RIP1 regulates an NF-kappaB-independent cell-death switch in TNF signaling. Curr. Biol. *17*, 418–424.

O'Donnell, M.A., Perez-Jimenez, E., Oberst, A., Ng, A., Massoumi, R., Xavier, R., Green, D.R., and Ting, A.T. (2011). Caspase 8 inhibits programmed necrosis by processing CYLD. Nat. Cell Biol. *13*, 1437–1442.

Oberst, A., Dillon, C.P., Weinlich, R., McCormick, L.L., Fitzgerald, P., Pop, C., Hakem, R., Salvesen, G.S., and Green, D.R. (2011). Catalytic activity of the caspase-8-FLIP(L) complex inhibits RIPK3-dependent necrosis. Nature *471*, 363–367.

Orme, M.H., Liccardi, G., Moderau, N., Feltham, R., Wicky-John, S., Tenev, T., Aram, L., Wilson, R., Bianchi, K., Morris, O., et al. (2016). The unconventional myosin CRINKLED and its mammalian orthologue MYO7A regulate caspases in their signalling roles. Nat. Commun. *7*, 10972.

Pasparakis, M., and Vandenabeele, P. (2015). Necroptosis and its role in inflammation. Nature *517*, 311–320.

Peltzer, N., Darding, M., and Walczak, H. (2016). Holding RIPK1 on the ubiquitin leash in TNFR1 signaling. Trends Cell Biol. *26*, 445–461.

Ran, F.A., Hsu, P.D., Wright, J., Agarwala, V., Scott, D.A., and Zhang, F. (2013). Genome engineering using the CRISPR-Cas9 system. Nat. Protoc. *8*, 2281–2308.

Sakurai, H. (2012). Targeting of TAK1 in inflammatory disorders and cancer. Trends Pharmacol. Sci. *33*, 522–530.

Schlicher, L., Wissler, M., Preiss, F., Brauns-Schubert, P., Jakob, C., Dumit, V., Borner, C., Dengjel, J., and Maurer, U. (2016). SPATA2 promotes CYLD activity and regulates TNF-induced NF-κB signaling and cell death. EMBO Rep. *17*, 1485–1497.

Silke, J. (2011). The regulation of TNF signalling: what a tangled web we weave. Curr. Opin. Immunol. *23*, 620–626.

Silke, J., Rickard, J.A., and Gerlic, M. (2015). The diverse role of RIP kinases in necroptosis and inflammation. Nat. Immunol. *16*, 689–697.

Söderberg, O., Gullberg, M., Jarvius, M., Ridderstråle, K., Leuchowius, K.J., Jarvius, J., Wester, K., Hydbring, P., Bahram, F., Larsson, L.G., and Landegren, U. (2006). Direct observation of individual endogenous protein complexes in situ by proximity ligation. Nat. Methods *3*, 995–1000.

Tenev, T., Bianchi, K., Darding, M., Broemer, M., Langlais, C., Wallberg, F., Zachariou, A., Lopez, J., MacFarlane, M., Cain, K., and Meier, P. (2011). The Ripoptosome, a signaling platform that assembles in response to genotoxic stress and loss of IAPs. Mol. Cell *43*, 432–448.

Ting, A.T., and Bertrand, M.J. (2016). More to Life than NF-κB in TNFR1 Signaling. Trends Immunol. *37*, 535–545.

Varfolomeev, E., Blankenship, J.W., Wayson, S.M., Fedorova, A.V., Kayagaki, N., Garg, P., Zobel, K., Dynek, J.N., Elliott, L.O., Wallweber, H.J., et al. (2007). IAP antagonists induce autoubiquitination of c-IAPs, NF-kappaB activation, and TNFalpha-dependent apoptosis. Cell *131*, 669–681.

Vince, J.E., Wong, W.W., Khan, N., Feltham, R., Chau, D., Ahmed, A.U., Benetatos, C.A., Chunduru, S.K., Condon, S.M., McKinlay, M., et al. (2007). IAP antagonists target cIAP1 to induce TNFalpha-dependent apoptosis. Cell *131*, 682–693.

Vince, J.E., Chau, D., Callus, B., Wong, W.W., Hawkins, C.J., Schneider, P., McKinlay, M., Benetatos, C.A., Condon, S.M., Chunduru, S.K., et al. (2008). TWEAK-FN14 signaling induces lysosomal degradation of a cIAP1-TRAF2 complex to sensitize tumor cells to TNFalpha. J. Cell Biol. *182*, 171–184.

Vlantis, K., Wullaert, A., Polykratis, A., Kondylis, V., Dannappel, M., Schwarzer, R., Welz, P., Corona, T., Walczak, H., Weih, F., et al. (2016). NEMO prevents RIP kinase 1-mediated epithelial cell death and chronic intestinal inflammation by NF-κB-dependent and -independent functions. Immunity *44*, 553–567.

Wagner, S.A., Satpathy, S., Beli, P., and Choudhary, C. (2016). SPATA2 links CYLD to the TNF-α receptor signaling complex and modulates the receptor signaling outcomes. EMBO J. *35*, 1868–1884.

Walczak, H. (2011). TNF and ubiquitin at the crossroads of gene activation, cell death, inflammation, and cancer. Immunol. Rev. *244*, 9–28.

Wang, M. (2017). ImmunoScore predicts gastric cancer postsurgical outcome. Lancet Oncol. *18*, e68.

Wang, L., Du, F., and Wang, X. (2008). TNF-alpha induces two distinct caspase-8 activation pathways. Cell *133*, 693–703.

Wong, W.W., Gentle, I.E., Nachbur, U., Anderton, H., Vaux, D.L., and Silke, J. (2010). RIPK1 is not essential for TNFR1-induced activation of NF-kappaB. Cell Death Differ. *17*, 482–487.

Yang, Y., Fang, S., Jensen, J.P., Weissman, A.M., and Ashwell, J.D. (2000). Ubiquitin protein ligase activity of IAPs and their degradation in proteasomes in response to apoptotic stimuli. Science *288*, 874–877.

STAR★METHODS

KEY RESOURCE TABLE

REAGENT or RESOURCE	SOURCE	IDENTIFIER
Antibodies		
Anti-Actin	Sigma	A5441
Anti-Caspase 8	Santa Cruz Biotechnology	sc-789
Anti-Caspase 8	Santa Cruz Biotechnology	sc-6136
Anti-cFLIP	Adipogene	AG-20B-0056
Anti-cIAP1	Enzo Life Sciences	ALX-803-335-C100
Anti-cIAP1	Enzo Life Sciences	ALX-803-335-C100
Anti-Cleaved Caspase 8	Cell Signaling	9429
Anti-ERK	Gift from Chris Marshall	N/A
Anti-FADD	Santa Cruz Biotechnology	sc-6036
Anti-FLAG [M2]	Sigma	F3165
Anti-HOIL	Gift from Henning Walczak	N/A
Anti-Hsp27	Santa Cruz Biotechnology	sc-13132
Anti-Hsp90	Santa Cruz Biotechnology	sc-7947
Anti-IkB α	Santa Cruz Biotechnology	sc-371
Anti-JNK	Santa Cruz Biotechnology	sc-571
Anti-MK2	Cell Signaling	3042
Anti-NEMO	Santa Cruz Biotechnology	sc-8330
Anti-P-ERK	Cell Signaling	9101
Anti-P-Hsp27	Santa Cruz Biotechnology	sc-166693
Anti-P-IkBα	Cell Signaling	2859
Anti-P-JNK	Cell Signaling	4668
Anti-P-MK2	Cell Signaling	3007
Anti-P-p38	Cell Signaling	9215
Anti-P-p65	Cell Signaling	3033
Anti-P-RIPK1 (S166) (rodent specific)	Cell Signaling	31122
Anti-P-RIPK1 (S320) (human)	Custom project; Thermo Fisher Scientific	N/A
Anti-P-RIPK1 (S321) (mouse)	Custom project; Thermo Fisher Scientific	N/A
Anti-p38	Cell Signaling	9212
Anti-p65	Cell Signaling	8242
Anti-RIPK1 (C-terminal)	BD Bioscience	610459
Anti-RIPK1 (N-terminal)	Cell Signaling	3493
Anti-RIPK3	Proscience	2283
Anti-Sharpin	ProteinTech	14626-1-AP
Anti-TAK1	Cell Signaling	4505
Anti-TNFR1	Abcam	19139
Anti-Tubulin	Sigma	T-9026
Anti-Ubiquitin	Dako	Z0458
Chemicals, Peptides, and Recombinant Proteins		
GSK'963 (RIPK1 inhibitor)	Gift from GSK	N/A
Compound A (Smac mimetic)	TetraLogic Pharmaceuticals	N/A
Necrostatin-1	BioVision	2263-5
FLAG-tagged hTNF	Enzo Life Sciences	ALX-804-034-C050
Human recombinant TNF	Enzo Life Sciences	ALX-522-008-C050
Mouse recombinant TNF	Enzo Life Sciences	ALX-522-009-C050

(*Continued on next page*)

Continued

REAGENT or RESOURCE	SOURCE	IDENTIFIER
BI605906 (IKK2 inhibitor)	MedChemExpress	HY-13019
TCPA-1 (IKK2 inhibitor)	Sigma	T-1452
(5Z)-7-Oxozeaenol (TAK1 inhibitor)	Tocris Bioscience	3604
BIRB 796 (p38 inhibitor)	LC Laboratories	D-2744
zVAD-FMK	Apex Bio	A1902
LY2228820 (p38 inhibitor)	Apex Bio	A5566
PF-3644022 (MK2 inhibitor)	Tocris Bioscience	4279
LPS	Invivogen	TLRL-PEKLPS
Tri-DAP	Invivogen	tlrl-tdap
PGN-EB	Invivogen	tlrl-pgneb
Human MK2 (active recombinant)	Thermo Scientific	PV3317
ATP	Thermo Scientific	R0441
Protein A/G agarose	Thermo Scientific	20423
Hoechst	Thermo Scientific	33342
Propidium iodide solution	Sigma	P4864
Ac-DEVD-AMC	Sigma	A1086
Halt Protease and phosphatase inhibitor	Thermo Scientific	78443
PR619	2B Scientific	SI9619
MTT reagent	Sigma	M5655
Murine Stem Cell factor (m-SCF)	Peprotech	250-03
Critical Commercial Assays		
Cell-Titer Glo Luminescent Cell Viability assay	Promega	G7571
Duolink In Situ Detection Reagents Green	Sigma	DUO92014
HiScribe T7 high yield RNA synthesis kit	NEB	E2040S
Deposited Data		
Raw data	Mendeley	http://dx.doi.org/10.17632/znt3g8r753.1
Experimental Models: Cell Lines		
Primary MEFs	In house	N/A
Primary BMDMs WT and $Mk2^{-/-}$	In house	N/A
Primary BMDMs $Mk2^{-/-}$	Gift from M. Gaestel	N/A
Primary MEF $Ripk1^{S321D/S321D}$	In house	N/A
Immortalized MEF $Ripk1^{K45A}$	In house	N/A
Primary MEF $Ripk1^{S321A/wt}$	In house	N/A
Immortalized MEFs WT and $Mk2^{-/-}$	Gift from Chris Marshall	N/A
BT549	ATCC	HTB-122
MDA-MB-468	In house	N/A
MDA-MB-231	In house	N/A
MLL-ENL WT, $Mk2^{-/-}$ and $Ripk1^{D138N}$	In house	N/A
Experimental Models: Organisms/Strains		
Mouse: C57BL/6 $Ripk1^{S321A/wt}$	In house	N/A
Mouse: C57BL/6 WT, $Mk2^{-/-}$ and $Ripk1^{D138N}$	In house	N/A
Mouse: C57BL/6 $Ripk1^{S321D/S321D}$	In house	N/A
Mouse: C57BL/6 Ly5.1 MLLENL	In house	N/A
Oligonucleotides		
RNA targeting RIPK1: GCTCGGGCGCCATGTAGTAG		N/A
RNA targeting TRADD: CCTGTTTGTGGAGTCCTCGC		N/A

(*Continued on next page*)

Continued		
REAGENT or RESOURCE	SOURCE	IDENTIFIER
RNA targeting TAK1: GTAAACACCAACTCATTGCG		N/A
RNA targeting IKK1: GAACCATGCCAATGTTGTAA		N/A
RNA targeting IKK2: ACCACCGCTCTCGGTTCCGG		N/A
RNA targeting NEMO:: GGCAGCAGATCAGGACGTAC		N/A
Recombinant DNA		
Cas9-plasmid	Addgene	41815 or 48138
pcDNA3	Thermo Scientific	V79020
pTRIPZ	GE Dharmacon	RHS4696
pTRIBZ	In house	(Tenev et al., 2011)
pCDNA5.5/FRT/TO vector	Thermo Scientific	V652020
px330 vector	Addgene	42230
Software and Algorithms		
CRISPR design	http://crispr.mit.edu	N/A
CRISPR design	http://www.addgene.org/crispr/church/	(Mali et al., 2013)
SAINT analysis	http://saint-apms.sourceforge.net/	(Choi et al., 2011)
Swiss-Prot	https://www.ebi.ac.uk/uniprot	N/A
Proteome Discoverer v1.4	Thermo Scientific	N/A
Image Lab V5.2.1.	Bio-Rad laboratories	N/A
Sequence alignment	https://benchling.com/	N/A
GraphPad Prism v6.0	http://www.graphpad.com/	N/A

CONTACT FOR REAGENT AND RESOURCE SHARING

Further information and requests for reagents may be directed to the Lead Contact, Pascal Meier (pmeier@icr.ac.uk).

EXPERIMENTAL MODEL AND SUBJECT DETAILS

Experimental Model

All animal procedures were conducted in accordance with the guidelines of The Walter and Eliza Hall Institute Animal Ethics Committee or European, national and institutional guidelines, and protocols were approved by local government authorities (Landesamt für Natur, Umwelt und Verbraucherschutz Nordrhein-Westfalen, Germany).

Generation of murine AML

All in vivo experiments were conducted in accordance with the guidelines of The Walter and Eliza Hall Institute Animal Ethics Committee. MLLENL retroviral construct were previously described (Lalaoui et al., 2016). Viral supernatants were produced in 293T cells by co-transfection of expression constructs and packaging plasmids. Fetal liver cells (E14.5) from WT $Mk2^{-/-}$ $Ripk1^{D138N}$ C57BL/6 Ly5.2 mice were infected with viral supernatant using the retronectin protocol. Transduced cells were cultured in alpha-MEM medium (Invitrogen) supplemented with 10% FCS, 2 mM L-glutamine, 100 ng/mL m-SCF, 10 ng/mL IL-6, 50 ng/ml TPO and 10 ng/ml Flt3 (WEHI). After two rounds of infection, cells were injected into sub-lethally γ-irradiated (7.5 Gy) C57BL/6 Ly5.1 mice. Mice were collected when disease was evident. Parameters used to determine leukemia were weight-loss, enlarge spleen, anemia, lethargy and hunched posture. Leukemic cells were obtained from bone marrow of sick mice. Cells were cultured at 37°C in a 10% CO_2 humidified atmosphere in IMDM media supplemented with 10% fetal calf serum and 2.5 ng/ml IL-3.

Mice generation

For the generation of *Ripk1-S321D* mice Cas9 mRNA (TriLink) together with the ssDNA repair oligo (IDT) and the short guide RNA (sgRNA) targeting the region surrounding S321 of the murine Ripk1 gene was microinjected into the pro-nucleus of fertilized oocytes obtained from C57BL/6 mice. The injected embryos were transferred to foster mothers and allowed to develop to term. Mutations in the genome of progeny were determined by analysis of genomic DNA using the T7 endonuclease I assay and sequencing. The

sequence of the ssDNA oligo used as a repair template for the *Ripk1-S321D* can be obtained upon request. sgRNA was generated by in vitro transcription, from the px330 vector containing the *Ripk1* targeting sequence.

Cell lines

MEFs, MDA-MB468, MDA-MB-231 and Flp-InT-Rex HEK293 cells were cultured in Dulbecco's modified Eagle's Medium (DMEM), BT549 were cultured in RPMI media. All media were supplemented with 10% Fetal Bovine Serum (FBS) and penicillin and streptomycin, under 10% CO_2. Immortalized *WT* and *Mk2*$^{-/-}$ MEFs were a kind gift from Chris Marshall.

METHOD DETAILS

Isolation of primary cells

Primary Mouse Embryonic Fibroblasts (MEFs) were generated from E13.5 embryos. After removing the placenta, yolk sac, head and the dark red organs, embryos were finely minced and digested for 20 min in 0.25% trypsin. Single cell suspension was then obtained by pipetting up and down the digested embryos. To generate Bone Marrow Derived Macrophages (BMDMs), bone marrow cells from tibia and femur of 2 month old mice were seeded in non-coated petri dishes and cultured for 6 days in Dulbecco's modified Eagle medium + 10% fetal bovine serum + 20% (v/v) L929 mouse fibroblast conditioned medium. To generate *Mk2*$^{-/-}$ BMDMs, lethally irradiated (9.5 Gy) WT C57BL/6 were reconstituted with *Mk2*$^{-/-}$ bone marrow, 8 weeks later bone marrow cells from tibia and femur from reconstituted mice were culture L929 conditioned medium for 6 days.

Constructs and transfection

For the generation of *Ripk1*$^{-/-}$ MEFs, primary *Ripk1*^{K45A} MEFs were infected with SV40T-expressing lentivirus for immortalization and subsequently infected with Cre recombinase-expressing lentivirus for *Ripk1* deletion as previously described (Berger et al., 2014). Human RIPK1 with deletion of DD (hRIPK1-ΔDD −1-581Aa) and mouse RIPK1 with deletion of DD (mRIPK1-ΔDD-1-567AA) were cloned into pTRIPZ or pTRIBZ and the respective cell lines were infected as described previously (Tenev et al., 2011).

Cell death and cell viability assays

5 x10^4 BMDMs or 8 x10^3 MEFs were seeded in 96 well plates and 24 hr later were treated as indicated for the indicated times. Hoechst (0.5 μg/ml) and PI (1 μg/ml) were added and the % of dead cells was measured using the CeligoS image cytometer (Nexcelon Bioscience). 5 x10^4 of MLL-ENL were seeded in 96 well plates and treated the same day as indicated for 24 hr. Cell death was analyzed by flow cytometry quantification of PI (2 μg/mL) uptake using a FACSCalibur (BD Biosciences).

Caspase activity assay (DEVDase)

Cells were plated in 24 well plates and treated as indicated. After treatment media was removed and plates were frozen at −80°C, to aid cell lysis. Next, plates were thawed and 50μl of 1% DISC lysis buffer (20 mM Tris-HCL pH 7.5, 150 mM NaCl, 2 mM EDTA, 1% Triton X-100, 10% glycerol) was added to each well, cells were scraped and lysates were left at Room Temperature for 15 min. 450 μl of DEVDase assay mix (20 μM Ac-DEVD-AMC (SIGMA), 1mM DTT, 25 mM HEPES pH 8.0) was added to the lysates NB: to measure all fractions cell lysates were not cleared). The plates were incubated at room temperature for up to 24 hr and DEVDase activity was read at 380nM excitation/460nM emission.

Generation of CRISPR cells

Guide RNAs were designed according to Zhang lab (Ran et al., 2013). MDA-MB-231 or 293FlpIn cells were transfected with pSpCas9-2A-GFP (Addgene) plasmid carrying gRNAs against human RIPK1-PM865, TRADD-16A35, TAK1-16A32, IKK1-16A25, IKK2 – 16A26 and NEMO-16A30 (sequence can be obtained upon request). 72 hr after transfection GFP positive clones were FACS sorted and single clones were screened for gene knockout.

In vitro kinase assay

L929 or HT29 cells were lysed in DISC buffer supplemented with protease inhibitors and clarified at 14,000 rpm at 4°C. Immunoprecipitations were performed using Protein A/G Plus agarose and rotated overnight at 4°C with anti-RIPK1 (C-terminal). Beads were washed 2x in wash buffer and 1x in kinase buffer (200 mM HEPES pH 8.0, 20 mM MgCl2, 5 mM EGTA, 0.05% Triton X-100). The kinase assay was performed in 30 μL kinase buffer containing 100 ng recombinant active MK2, 30 μM ATP and MK2 inhibitor where indicated. Beads were incubated for 30 min at 30°C, and reactions were halted by addition of 30 μL 2x SDS sample buffer. Samples were boiled and the results visualized by Western Blot.

Tube pull-down

Cells were lysed in DISC lysis buffer supplemented with protease inhibitors, 1 mM DTT, PR619 (10 μM) and GST-TUBE (50 μg/ml; 50 μg TUBE/mg protein lysate). Cell lysates were rotated at 4°C for 20 min then clarified at 4°C at 14,000 rpm for 10 min. 20 μL

GST beads were added and immunoprecipitations were performed overnight. Beads were washed 4x in wash buffer (50 mM Tris pH 7.5, 150 mM NaCl, 0.1% Triton X-100, and 5% glycerol) + PR619 (10 μM), and bound proteins eluted by boiling in 50 μ l 1x SDS loading dye.

UbiCRest

The UbiCRest analysis with linkage selective DUBs was performed essentially as described in Hospenthal et al. (2015). Briefly, the released fraction (see complex-I purification) was incubated with 1 μM OTULIN, 0.2 μM OTUD1, 1 μM CEZANNE, 0.2 μM OTUB1, 1.5 μM USP21. The reaction was conducted in the presence of 1 mM DTT for 30 min at 37°C. Reactions were stopped with SDS sample buffer, and the ubiquitylation status analyzed by western blotting.

Complex-I/II Purification

Cells were seeded in 15 cm dishes and treated as indicated with 3x FLAG-hTNF (5 μg/ml). Media was removed and plates were washed with ice cold PBS. Plates were frozen at −80°C. Plates were thawed on ice and cells were lysed in 1% Triton X-100 lysis buffer (30 mM Tris-HCl pH 7.4, 120 mM NaCl, 2 mM EDTA, 2 mM KCl, 10% glycerol and 1% Triton X-100) + protease inhibitors and PR619 (10 μM). Cell lysates were rotated at 4°C for 20 min then clarified at 4°C at 14,000 rpm for 30 min. Proteins were immunoprecipitated with 20 μL of α-FLAG M2 beads (SIGMA) with rotation overnight at 4°C. For the 0 hr sample 5 μg/ml of FLAG-TNF was added post-lysis. Beads were washed 4x washes in lysis buffer and samples eluted by boiling in 60 μL 1x SDS loading dye. For complex-II purification MEFs and BMDMs were seeded in 10 and 15 cm dishes respectively and treated as indicated in Figure legend. Cells were lysed on ice as above. Cell lysates were rotated at 4°C for 20 min then clarified at 4°C at 14,000 rpm for 10 min. 20 μL of protein G Sepharose, blocked for 1 hr with lysis buffer containing 1% BSA, were bound with FADD antibody [1.5 μg antibody/mg lysate] and incubated with protein lysates 4 hr at 4°C. Beads were washed 4x in lysis buffer and samples eluted by boiling in 60 μL 1x SDS loading dye.

Proximity ligation assay (PLA)

PLA was performed according to the manufacturer's protocol using the Duolink Detection Kit (SIGMA). Cells were examined with a confocal microscope (objective x 40, Zeiss LSM 710).

QUANTIFICATION AND STATISTICAL ANALYSIS

Statistical analysis was performed using GraphPad Prism V6.0. Unless otherwise specified, data are presented as mean ± SEM. Comparisons were performed with a Student's t test whose values are represented in the figures as *$p < 0.05$, **$p < 0.01$, and ***$p < 0.001$.

DATA AND SOFTWARE AVAILABILITY

Raw data have been deposited to Mendeley Data and are available at http://dx.doi.org/10.17632/znt3g8r753.1.

Molecular Cell, Volume *66*

Supplemental Information

MK2 Phosphorylates RIPK1 to Prevent TNF-Induced Cell Death

Isabel Jaco, Alessandro Annibaldi, Najoua Lalaoui, Rebecca Wilson, Tencho Tenev, Lucie Laurien, Chun Kim, Kunzah Jamal, Sidonie Wicky John, Gianmaria Liccardi, Diep Chau, James M. Murphy, Gabriela Brumatti, Rebecca Feltham, Manolis Pasparakis, John Silke, and Pascal Meier

Supplementary Figures

Figure S1

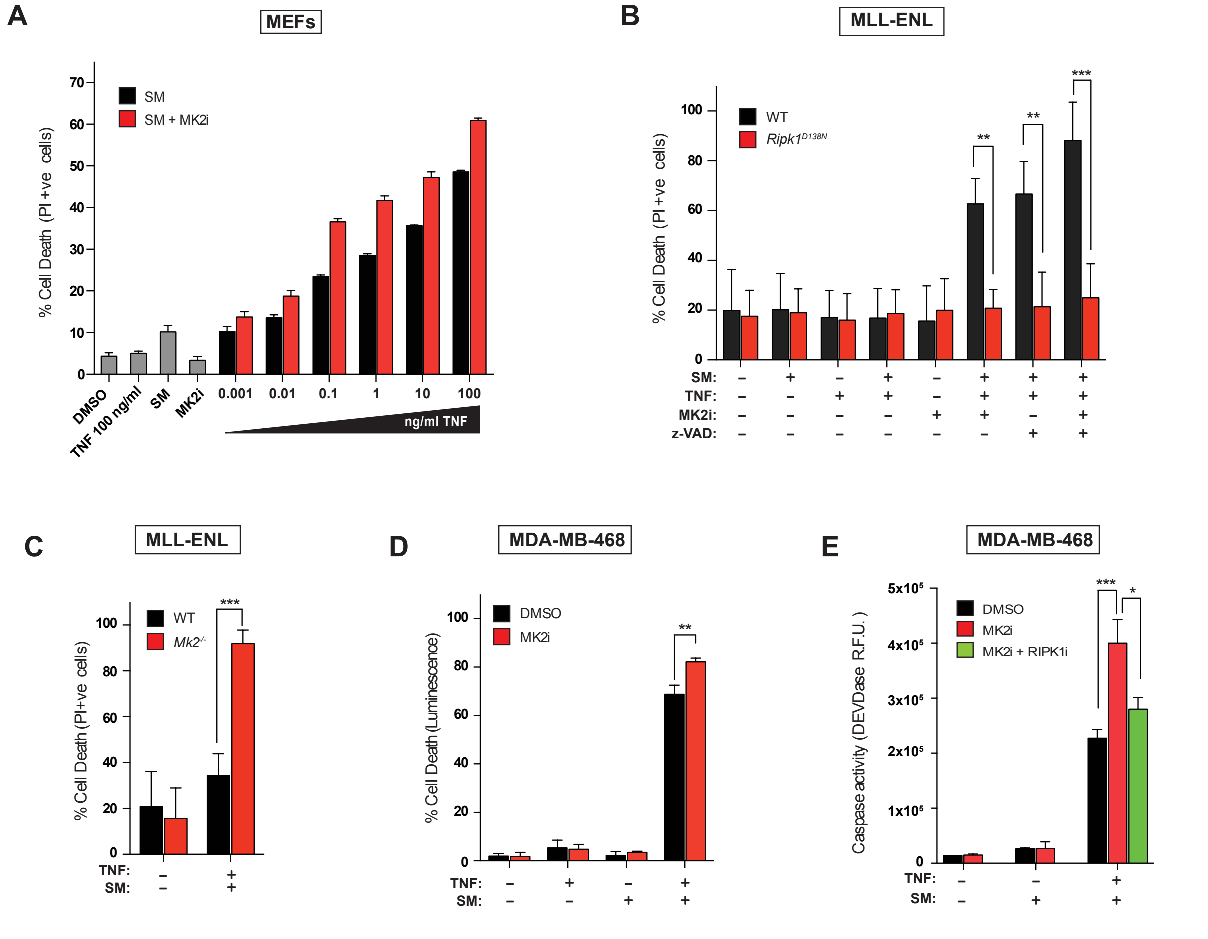

Figure S1. MK2 protects from TNF-induced cell death, Related to Figure 1

(A) Quantification of PI positive cells of primary MEFs treated with the indicated agents. Cells were pre-treated with MK2i (1 μM). **(B)** Quantification of PI positive cells of WT and *Ripk1*^{D138N} MLL-ENL cells. Cells were treated with the indicated agents for 24 hrs. TNF (10 ng/ml), SM (25 nM), MK2i (2 μM). **(C)** Quantification of PI positive cells of WT and *Mk2*$^{-/-}$ MLL-ENL cells, treated with the indicated agents for 24 hrs. TNF (10 ng/ml), SM (25 nM). **(D)** Quantification of cell viability in MDA-MB-468 cells, treated with the indicated agents. Cells were pre-treated with MK2i (1 μM). **(E)** DEVDase activity analysis of MDA-MB-468 cells left untreated or treated with the indicated agents for 5 hrs. Cells were pre-treated with MK2i (1 μM) and RIPK1i (100 nM) for 30 min. Graphs show mean ± SEM, n = 3 independent biological repeats. *$p < 0.05$, **$p < 0.01$, and ***$p < 0.001$.

Figure S2

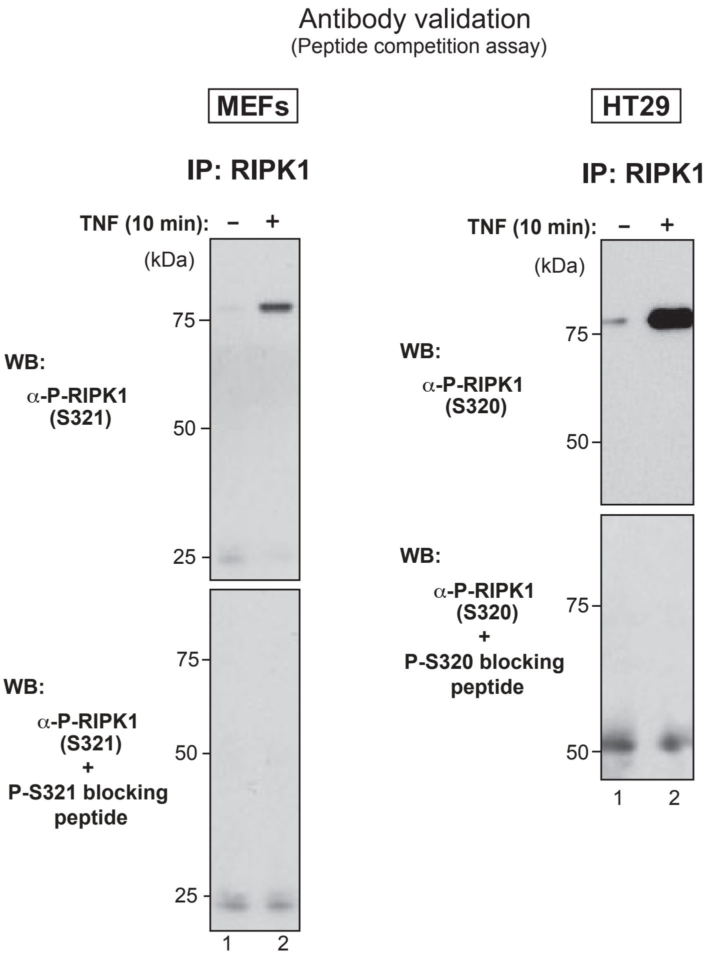

Figure S2. Antibody validation, Related to Figure 2

Western blot analysis of RIPK1 immunoprecipitates from MEFs or HT29 cells using the indicated antibodies. Cells were stimulated with TNF (10 ng/ml) for 10 min. Antibodies were either left untreated or pre-incubated with blocking P-peptide, against which these antibodies were raised against.

Figure S3

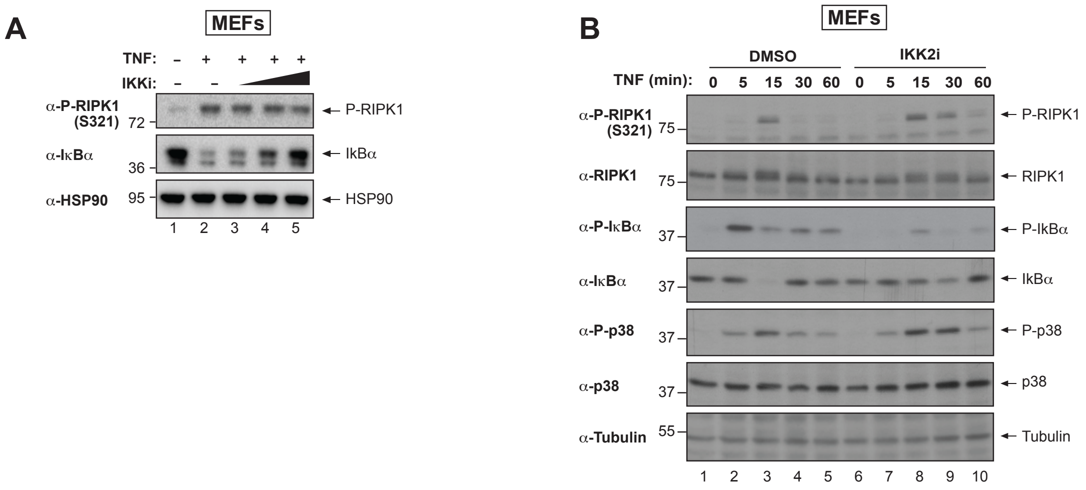

Figure S3. MK2 phosphorylates RIPK1 at S320/S321 in response to TNF stimulation, Related to Figure 3

(A) Western blot analysis of cell lysates from primary MEFs using the indicated antibodies. Cells were pre-treated with increasing concentrations of IKK2i (TCPA-1, 0.2, 1 and 5 μM) for 1 h, followed by stimulation with TNF (10 ng/ml) for 10 min. **(B)** Western blot analysis of cell lysates from primary MEFs using the indicated antibodies. Cells were pre-treated with IKK2i (BI605906, 10 μM) for 1 hr, followed by stimulation with TNF (10 ng/ml) for the indicated times.

Figure S4

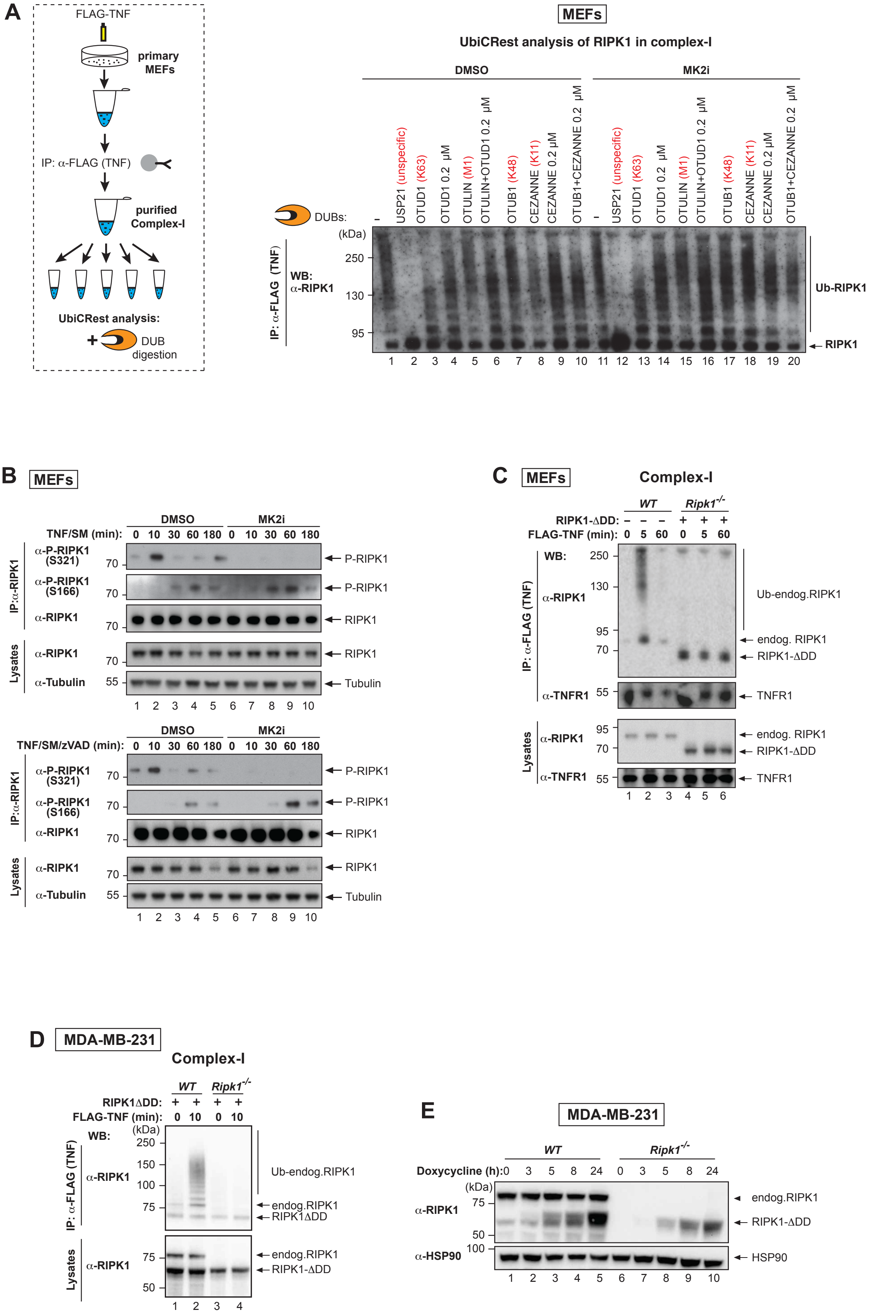
A
FLAG-TNF
primary MEFs
IP: α-FLAG (TNF)
purified Complex-I
UbiCRest analysis:
DUB digestion
MEFs
UbiCRest analysis of RIPK1 in complex-I
DMSO
MK2i
DUBs:
USP21 (unspecific)
OTUD1 (K63)
OTUD1 0.2 μM
OTULIN (M1)
OTULIN+OTUD1 0.2 μM
OTUB1 (K48)
CEZANNE (K11)
CEZANNE 0.2 μM
OTUB1+CEZANNE 0.2 μM
(kDa)
250
130
95
IP: α-FLAG (TNF)
WB: α-RIPK1
Ub-RIPK1
RIPK1
1 2 3 4 5 6 7 8 9 10 11 12 13 14 15 16 17 18 19 20
B
MEFs
TNF/SM (min): 0 10 30 60 180 0 10 30 60 180
α-P-RIPK1 (S321)
α-P-RIPK1 (S166)
α-RIPK1
α-Tubulin
IP:α-RIPK1
Lysates
P-RIPK1
RIPK1
Tubulin
TNF/SM/zVAD (min): 0 10 30 60 180 0 10 30 60 180
1 2 3 4 5 6 7 8 9 10
C
MEFs
Complex-I
WT
Ripk1-/-
RIPK1-ΔDD: – – – + + +
FLAG-TNF (min): 0 5 60 0 5 60
WB:
α-RIPK1
α-TNFR1
Ub-endog.RIPK1
endog. RIPK1
RIPK1-ΔDD
TNFR1
1 2 3 4 5 6
D
MDA-MB-231
Complex-I
RIPK1ΔDD: + + + +
FLAG-TNF (min): 0 10 0 10
Ub-endog.RIPK1
endog.RIPK1
RIPK1ΔDD
RIPK1-ΔDD
1 2 3 4
E
MDA-MB-231
Doxycycline (h): 0 3 5 8 24 0 3 5 8 24
α-RIPK1
α-HSP90
endog.RIPK1
RIPK1-ΔDD
HSP90
1 2 3 4 5 6 7 8 9 10

Figure S4. MK2-dependent phosphorylation of RIPK1 does not affect NF-κB signalling, Related to Figure 4

(A) UbiCRest analysis of ubiquitylated RIPK1 in complex-I. Complex-I was purified from primary WT MEFs using FLAG-mTNF as affinity reagent. Immuno-complexes were then subjected to UbiCRest analysis using the indicated panel of DUBs followed by Western blot analysis for RIPK1. **(B)** Immuno-precipitation of RIPK1 from DMSO and MK2i-treated MEFs (1 μM). The presence of the indicated proteins was evaluated by western blot. Cells were treated with the indicated agents for the indicated time points. **(C,D)** TNF-induced complex-I immuno-precipitation using FLAG-mTNF or FLAG-hTNF (1 μg /ml) as an affinity reagent from lysates of WT and *Ripk1*$^{-/-}$ MEFs (C) and MDA-MB-231 (D) cells stably expressing an inducible form of RIPK1-ΔDD. Western blot analysis with the indicated antibodies is shown. **(E)** Western blot analysis of cell lysates of WT and *Ripk1*$^{-/-}$ MDA-MB-231 cells stably expressing RIPK1-ΔDD. Expression of RIPK1-ΔDD was induced by Doxycycline (1 μg/ml), and the presence and phosphorylation status of the indicated proteins was evaluated.

Figure S5

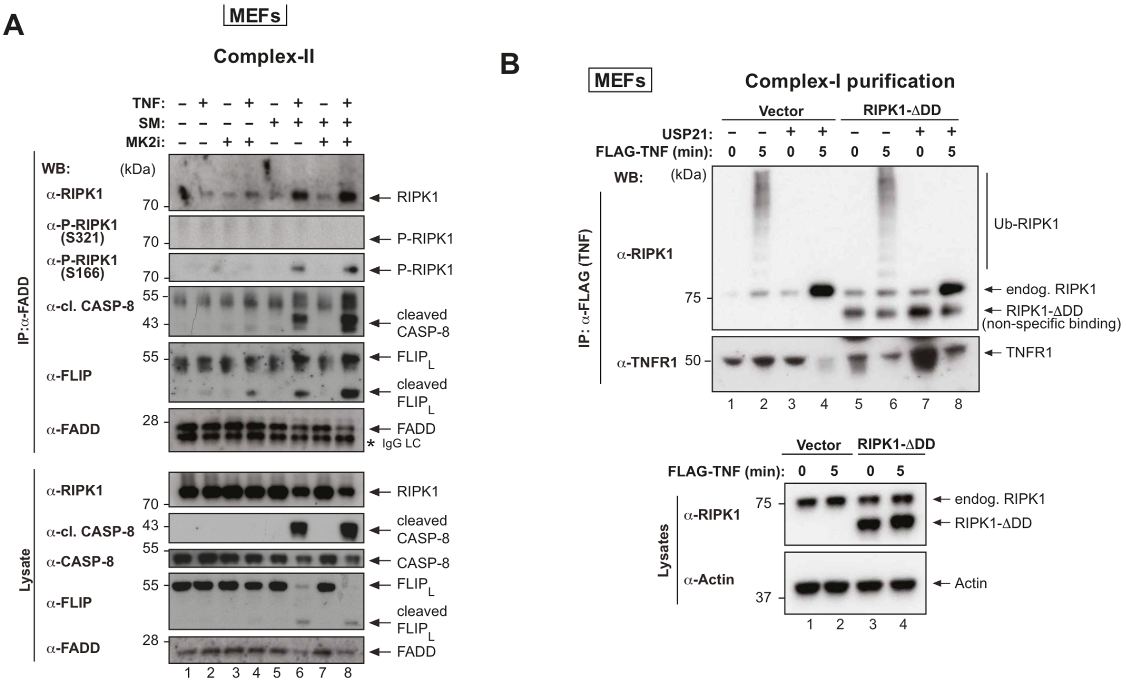

Figure S5. RIPK1-ΔDD is not recruited to TNF complex-I, Related to Figure 5

(A) TNF-induced complex-II was immuno-precipitated with anti-FADD from MEF lysates. Cells were treated with TSZ for 3 hrs ± MK2i (2 μM). **(B)** TNF-induced complex-I immuno-precipitation using FLAG-mTNF (1 μg/ml) as an affinity reagent from lysates of WT MEFs stably expressing and inducible form of RIPK1-ΔDD. Western blot analysis with the indicated proteins is shown.

Figure S6

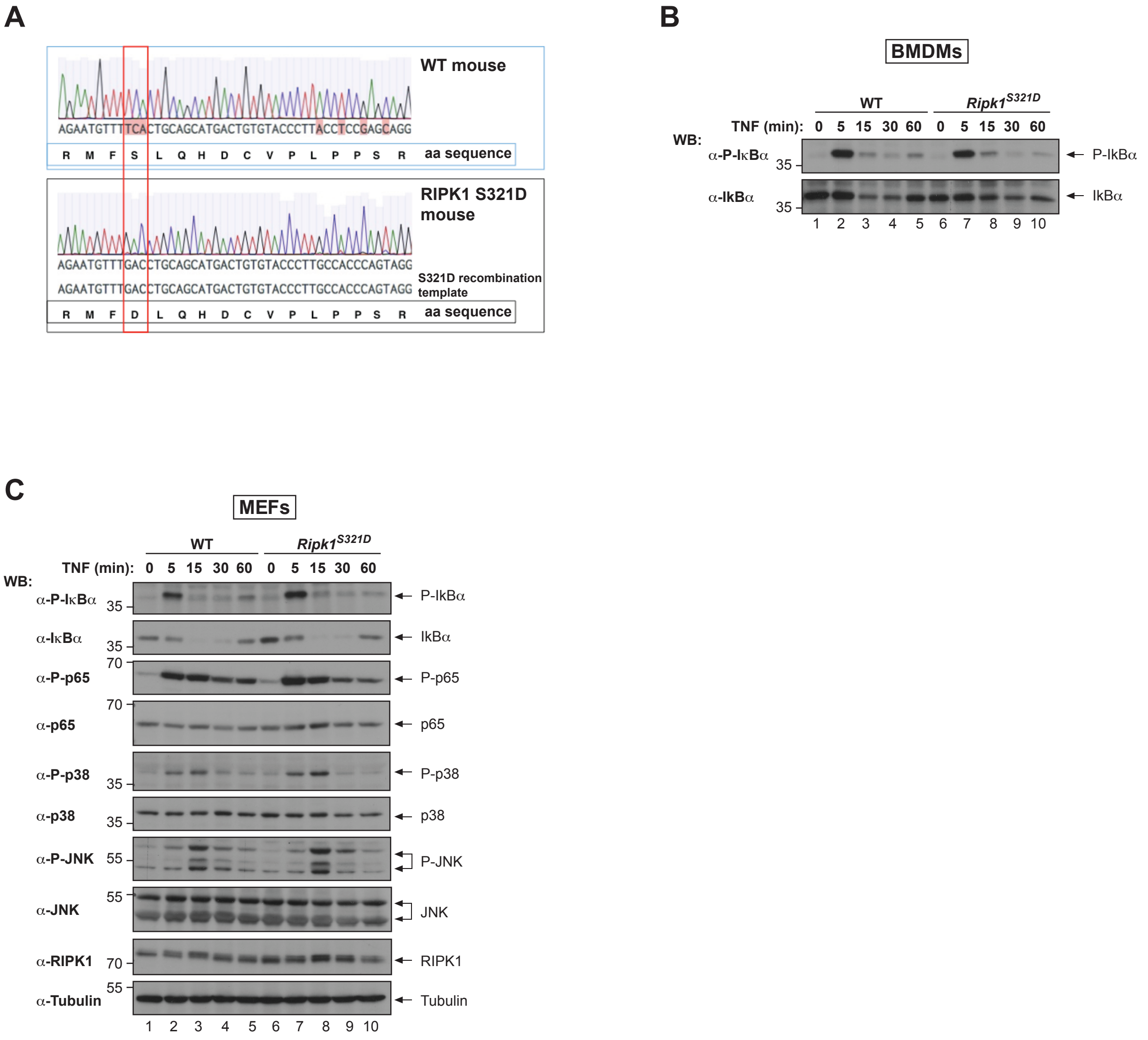

Figure S6. MK2-dependent phosphorylation of RIPK1 at S321 protects cells from TNF induced cell death, Related to Figure 6

(A) Sequence of the *Ripk1* gene in WT and *Ripk1*$^{S321D/S321D}$ mice. In addition to the TCA to GAC mutation changing S at position 321 to D, silent mutations were introduced in the PAM sequence to protect the mutated allele from Cas9-mediated cleavage. **(B, C)** Western blot analysis of primary BMDMs (B) and MEFs (C) isolated from WT and *Ripk1*^{S321D} mice using the indicated antibodies. Cells were treated with TNF (10 ng/ml) for the indicated time points.
